# Fabrication of Bioactive *Helix aspersa* Extract-Loaded Chitosan-Based Bilayer Wound Dressings for Skin Tissue Regeneration

**DOI:** 10.1021/acsomega.4c04345

**Published:** 2024-11-26

**Authors:** Merve Perpelek, Sedef Tamburaci, Ahmet Karakasli, Funda Tihminlioglu

**Affiliations:** † Department of Biomechanics, Dokuz Eylul University, Balcova, İzmir 35330, Turkey; ‡ Department of Chemical Engineering, İzmir Institute of Technology, Urla, İzmir 35430, Turkey; § Department of Orthopedics and Traumatology, Dokuz Eylul University, Balcova, İzmir 35330, Turkey

## Abstract

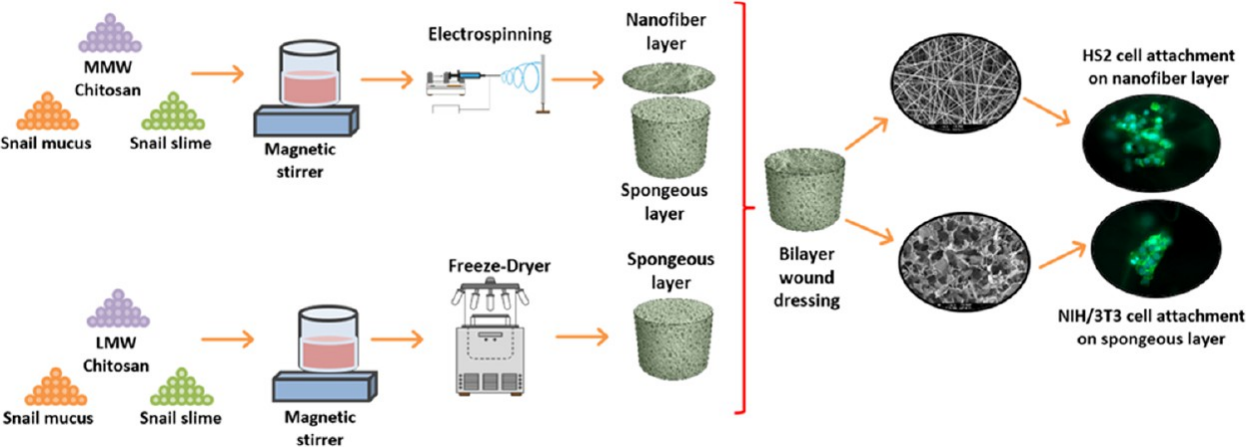

In recent years, there has been a notable shift toward exploring plant and animal extracts for the fabrication of tissue engineering structures that seamlessly integrate with the human body, providing both biological compatibility and physical reinforcement. In this particular investigation, we synthesized bilayer wound dressings by incorporating snail (*Helix aspersa*) secretions, comprising mucus and slime, into chitosan matrices via lyophilization and electrospinning methodologies. A nanofiber layer was integrated on top of the porous structure to mimic the epidermal layer for keratinocyte activity as well as acting as an antibacterial barrier against possible infection, whereas a porous structure was designed to mimic the dermal microenvironment for fibroblast activity. Comprehensive assessments encompassing physical characterization, antimicrobial efficacy, in vitro bioactivity, and wound healing potential were conducted on these bilayer dressings. Our findings revealed that the mucus and slime extract loading significantly altered the morphology in terms of nanofiber diameter and average pore size. Snail extracts loaded on a nanofiber layer of bilayer dressings showed slight antimicrobial activity against *Staphylococcus epidermidis* and *Escherichia coli*. An in vitro release study of slime extract loaded in the nanofiber layer indicated that both groups 1 and 2 showed a burst release up to 6 h, and a sustained release was observed up to 96 h for group 1, whereas slime extract release from group 2 continued up to 72 h. In vitro bioactivity assays unveiled the favorable impact of mucus and slime extracts on NIH/3T3 fibroblast and HS2 keratinocyte cell attachment, proliferation, and glycosaminoglycan synthesis. Furthermore, our investigations utilizing the in vitro scratch assay showcased the proliferative and migratory effects of mucus and slime extracts on skin cells. Collectively, our results underscore the promising prospects of bioactive snail secretion-loaded chitosan constructs for facilitating skin regeneration and advancing wound healing therapies.

## 1 Introduction

The skin, the body’s largest organ after the skeletal system, serves as a crucial barrier against external factors, highlighting the significance of recent advancements in skin tissue regeneration and transplantation for clinical applications. Skin injuries, stemming from trauma, accidents, medical conditions (such as diabetic ulcers), or other causes, such as burns, present critical challenges.^1^ While minor wounds often heal spontaneously, larger wounds pose significant risks, including infection, prolonged hospitalization, increased morbidity, chronic wound formation, organ failure, and mortality.^2,3^ Wound healing, a multifaceted process involving various growth factors, cell types, and cytokines,^4,5^ progresses through hemostasis, inflammation, cell proliferation, and remodeling phases.^3^ Traditional approaches to wound care, encompassing surgery, allogeneic skin grafts, thermal therapies, and the use of antioxidants and anti-inflammatory agents, entail inherent risks such as infection, donor incompatibility, and immunogenic reactions.^2,3,6^

In recent years, researchers have increasingly turned to tissue engineering to mitigate these drawbacks. Scaffolds fabricated from biomaterials have shown promising outcomes in tissue regeneration. An ideal scaffold should mimic the skin’s biological, physical, and chemical properties with appropriate pore size to allow oxygen and nutrient diffusion, fostering the growth and proliferation of skin cells while possessing mechanical characteristics akin to native tissue. The skin is composed of two layers, namely, the epidermis, which is the outermost layer containing keratinocyte cells, and the dermis layer, which lies under the epidermis consisting of collagen, hyaluronic acid, elastin, extracellular matrix (ECM), glycosaminoglycan (GAG), proteoglycans, and fibroblast cells.^7^ Thus, recent studies have focused on multilayered wound dressings composed of different layers that mimic skin tissue in terms of structure and morphology.^8−10^

Natural polymers, prized for their biocompatibility, biodegradability, and formability, are commonly employed in scaffold design.^11,12^ Natural polymers play a significant role in various wound healing processes due to these properties. Their biocompatibility and antimicrobial characteristics prevent any immunogenic reaction in the tissue, and being biodegradable, they accelerate the healing process when applied to the tissue. Additionally, they allow nutrient diffusion, which triggers fibroblast proliferation and contributes to collagen synthesis necessary for wound healing.^13^ However, their poor mechanical properties necessitate the incorporation of reinforcing bioactive agents or nanofibers within the polymer matrix.^10,14,15^ Chitosan (CHI), a frequently utilized biopolymer in tissue engineering, boasts biocompatibility, biodegradability, structural similarities to the extracellular matrix (e.g., glycosaminoglycan (GAG)), and antibacterial properties.^16,17^

In multilayered wound dressing designs, the combination of natural polymers with animal- or plant-based bioactive agents has come into prominence to support wound healing as well as ensure antimicrobial activity for possible infection at the defect site.^9,10,15^ Among animal-based sources, *Helix aspersa*, known as garden snail, has two secretions, namely, mucus (M) and slime (S), that have many ingredients convenient to the human body, such as collagen, allantoin, elastin, glycolic acid, vitamins A, E, and C, and GAG.^18,19^ Slime is the secretion that snails leave behind during their movement, whereas mucus is the adhesive secretion produced from the epithelium of the dorsal and lateral foot of snails.^19^ In our previous studies, we incorporated *H. aspersa* mucus and slime extracts in a CHI matrix and investigated the effects of snail extracts on biomaterials with physical, chemical, and in vitro bioactivity of the CHI matrix for bone and cartilage regeneration. Our results revealed that mucus and slime extract loading enhanced the mechanical properties and biodegradability of single layer and gradient CHI scaffolds, and extracts did not show any cytotoxic effect on cells, inducing in vitro osteogenic and chondrogenic activity. The similarity of extract content with the ECM matrix components ensures convenient microenvironment for cell attachment and proliferation. In addition, mucus and slime extracts showed antimicrobial activity for *Staphylococcus epidermidis* and *Pseudomonas fluorescens* bacteria.^20,21^ In the literature, pure *H. aspersa* snail secretions have been investigated for wound healing applications. Gubitosa et al. fabricated snail (*H. aspersa*) slime (SS)-loaded gold (Au) nanoparticles (NPs) and investigated their in vitro wound healing potential as well as their anti-inflammatory properties. Results demonstrated that Au-NPSS increased wound closure and modulated inflammatory response by reducing IL1-β and IL-6 cytokine levels.^5^ In addition, other studies indicated that *H. aspersamuller* slime^22^ and *H. aspersa* mucus^23^ showed significant wound healing capacity by inducing fibroblast proliferation^24^ as well as showing antimicrobial and anti-inflammatory properties. Zamudio et al. fabricated gelatin/chitosan-based scaffolds with aloe vera and *H. aspersamuller* mucus and investigated the wound repair capacity of scaffolds through in vivo experiments only. Their findings suggest that these mucus and aloe vera-infused scaffolds hold considerable promise as biomaterials for facilitating wound healing.^25^ However, there is a paucity of literature on the wound healing potential of biomaterials incorporating snail secretions, indicating a need for further research. Future investigations should focus on developing wound dressings that closely mimic skin layers while demonstrating the bioactive properties of snail secretions.

The objective of this study was to fabricate bioactive chitosan-based bilayer wound dressings loaded with snail secretion extracts, comprising both mucus and slime, to enhance the physical characteristics and cellular bioactivity of fibroblast and keratinocyte cells for skin tissue regeneration as well as show a slight antimicrobial effect against possible infection risk at the defect site. A nanofiber layer was designed to mimic the epidermis as well as provide the release of antimicrobial slime extract, whereas a sponge layer was designed to mimic the dermal layer for fibroblast proliferation and ECM formation. Additionally, the synergistic effects of mucus and slime extracts within the chitosan matrix were explored by integrating them in nanofiber and sponge layers. Bilayer wound dressings were prepared via the integration of layers fabricated with lyophilization and electrospinning methods. Physical characterization and in vitro bioactivity analysis were conducted to assess the properties of the bilayer wound dressings. Specifically, in vitro bioactivity studies utilizing keratinocyte (HS2) and fibroblast (NIH/3T3) cells evaluated the cytotoxicity, proliferation, wound healing, and biomarker secretion associated with skin tissue regeneration, while the antimicrobial properties against *S. epidermidis* and *Escherichia coli* bacteria were assessed for both the extracts and bilayer structures.

## 2 Materials and Methods

### 2.1 Materials

Low-molecular-weight chitosan powder (Sigma-Aldrich), medium-molecular-weight chitosan powder (Sigma-Aldrich), genipin (Wako), poly(ethylene oxide) (Sigma-Aldrich), *H. aspersa* mucus extract (Medical grade, Xi’an SR Bio-Engineering Co., Ltd.), and *H. aspersa* slime extract (Pharmaceutical grade, Xi’an Nate Biological Technology Co., Ltd.) were used for bilayer wound dressing fabrication. Protein adsorption was detected with the Pierce BCA protein assay (Thermo Fisher Scientific) and bovine serum albumin (BSA; Sigma-Aldrich). Dulbecco’s modified Eagle medium (High glucose, Serox), penicillin–streptomycin (Serox), l-glutamine (Serox), fetal bovine serum (FBS, Serox), WST-1 assay (BioVision Inc.), CCK-8 Kit (Abbkine), Alexa Fluor 488 (Thermo Fisher Scientific), DAPI (Thermo Fisher Scientific), proteoglycan assay (Amsbio, AMS Biotechnology), hydroxyproline colorimetric assay kit (Elabscience), Safranin-O staining (ScienCell 8348), and Type I collagen ELISA assay kit (Sunlog Chemicals) were used in in vitro biocompatibility, in vitro bioactivity, and wound healing studies.

### 2.2 Fabrication of Chitosan (CHI), Chitosan–Mucus (CHI–M), and Synergic (SYN) Porous Layer

To prepare the control group, low-molecular-weight (LMW) chitosan (CHI) at a concentration of 1% (w/v) was dissolved in a 1% (v/v) acetic acid solution. The mucus-incorporated group (CHI–M) was formulated by dissolving 1% (w/v) CHI in 80 mL of acetic acid and dispersing mucus powder at a concentration of 1% (w/v) in 20 mL of acetic acid solution. For the synergic group (SYN), the spongeous layer was prepared by dissolving 1% (w/v) CHI in 80 mL of acetic acid and dispersing a mixture of mucus and slime powder, each at a concentration of 0.5% (w/v), in 20 mL of an acetic acid solution. Subsequently, the CHI and extract solutions were blended using a magnetic stirrer and left to mix overnight. Finally, the solutions were dispensed into 24-well plates, frozen at −20 °C for 24 h, and subjected to lyophilization at −46 °C under a pressure of 0.018 mbar to fabricate the porous bottom layer of the bilayer wound dressing.

### 2.3 Fabrication of Chitosan (CHI), Chitosan–Slime (CHI–S), and Synergic (SYN) Nanofiber Layer

**Figure 1:**
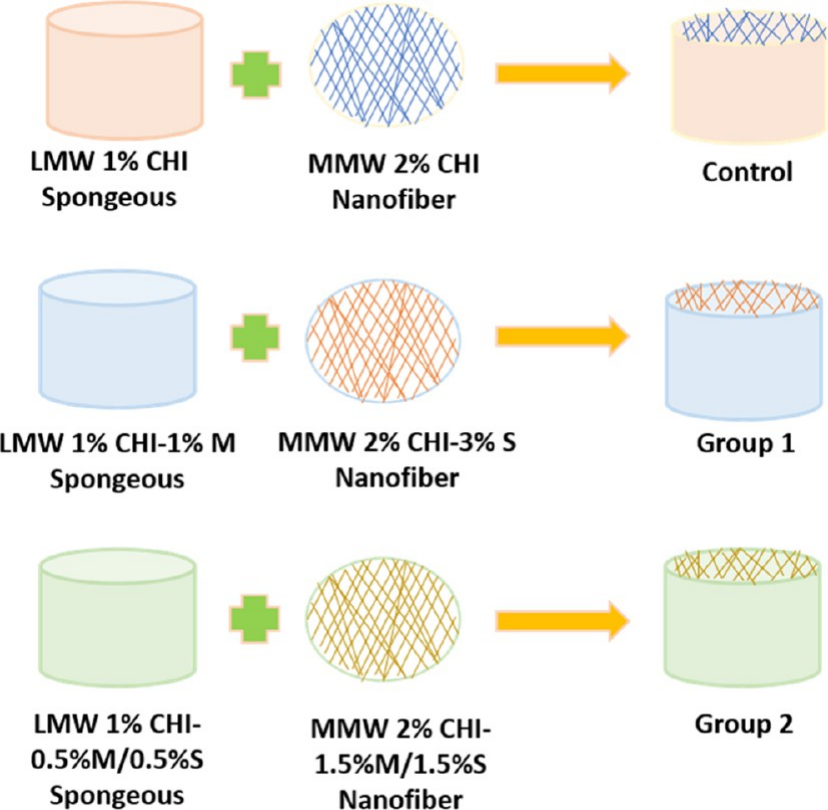
Schematic view of preparation of bilayer wound dressing group compositions.

Nanofiber layers were fabricated via the electrospinning method, employing a 10 cm distance between the spinneret and the collector, a voltage of 20 kV, and a flow rate of 2 mL/h. In the electrospinning process, poly(ethylene oxide) (PEO) served as a plasticizer, while genipin acted as a cross-linker in the chitosan solution. For the medium-molecular-weight (MMW) CHI group, a solution comprising 2% (w/v) CHI, 1% (w/v) genipin, and 0.5% (w/v) poly(ethylene oxide) was dissolved in acetic acid (70% v/v). In the CHI–S group, the solution consisted of 2% (w/v) CHI, 1% (w/v) genipin, and 0.5% (w/v) poly(ethylene oxide) dissolved in 80 mL of acetic acid solution, with slime powder (3% S) dispersed in an additional 20 mL of acetic acid solution. Similarly, for the synergic (SYN) group, a solution containing 2% (w/v) CHI, 1% (w/v) genipin, and 0.5% (w/v) poly(ethylene oxide) was prepared in 80 mL of acetic acid solution, while a mixture of mucus and slime powder (1.5% M and 1.5% S) was dispersed in an additional 20 mL of acetic acid solution. Subsequently, the CHI solutions and extract solutions were blended using a magnetic stirrer and left to mix overnight. Finally, the solutions were utilized for the electrospinning process, and (M)- and (S)-loaded nanofibers were coated onto nanocomposite sponges by immobilizing on the sample collector during the electrospinning process (Figure 1 and Table 1).

**Table 1:** Contents of the Upper and Bottom Layers for Bilayer Wound Dressings

groups	nanofiber (upper) layer	spongeous (bottom) layer
control	2% MMW chitosan (CHI)	1% LMW chitosan (CHI)
group 1	2% MMW chitosan (CHI)–3% slime (S)	1% LMW chitosan (CHI)–1% mucus (M)
group 2	2% MMW chitosan (CHI)–1.5% mucus (M)/1.5% slime (S)	1% LMW chitosan (CHI)–0.5% mucus (M)/0.5% slime (S)

### 2.4 Characterization of the Bilayer Wound Dressings

#### 2.4.1 Scanning Electron Microscopy (SEM) Analysis: Stereomicroscope Examination

The morphology of the bilayer wound dressing was visualized with scanning electron microscopy to observe the effect of mucus and slime extract on the pore structure. The upper and bottom layers and the cross-sectional sample of the bilayer wound dressing were visualized separately. Samples were coated with a thin gold layer under argon gas before SEM analysis (Quanta FEG). The average pore size, nanofiber diameter, and layer thickness of the bilayer wound dressing were calculated using ImageJ software.

The stability of the bilayer wound dressings was examined as previously described.^26^ The dry bilayer scaffolds and the wet samples that were incubated in 1× PBS solution (at 37 °C) for 7 days were visualized with a stereomicroscope to observe the integrity and stability of spongeous and nanofiber layers of groups.

#### 2.4.2 Open Porosity Measurement by the Liquid Displacement Method

The porosity of the samples was determined with the liquid displacement method for the spongeous layer. The samples (*n* = 3) were placed in a tube containing ethanol solution (*V*_1_). The total volume of the spongeous sample with ethanol was noted as *V*_2_, and elevation levels of ethanol were determined. Specimens were taken from the tubes, and decreased ethanol levels were measured (*V*_3_). Open porosity % was calculated with the equation defined as follows.
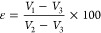
1

#### 2.4.3 Water Absorption Capacity

The swelling behavior of the spongeous layer and bilayer wound dressing (*n* = 3) scaffolds was evaluated as previously described in our study.^20^ Water absorption capacity was measured at 24 and 48 h, and swelling % was calculated with the equation defined as follows.
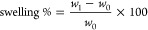
2

#### 2.4.4 Mechanical Properties

The mechanical properties of spongeous layers (*n* = 5) were demonstrated using a TA-XT Plus Texture Analyzer with the compression test according to the ASTM D 5024–95a standard. Compression tests were performed with a 5 kN load and 5 mm min^–1^ speed at 75% strain. Results were calculated as the Young modulus.

#### 2.4.5 Protein Adsorption on the Material Surface

The protein adsorption of the samples (*n* = 3) was determined using the BCA (Pierce, Rockford, IL) assay and 0.1% BSA (bovine serum albumin) as the reference protein solution previously described.^27^

#### 2.4.6 In Vitro Release Study

In vitro release studies were carried out with an antimicrobial slime-loaded nanofiber layer of bilayer wound dressings. Before experiments, spectrophotometric scanning was carried out between 100 and 400 nm for the assessment of bioactive component peaks in slime extracts. The maximum UV–vis peak was obtained at a 280 nm wavelength. Extract-loaded samples were dissolved in 1% acetic acid solutions for 1 h to destroy the polymeric structure. The amount of slime-released media from the nanofiber layer of wound dressings was measured at 280 nm with a UV spectrophotometer (Varioskan). The encapsulation efficiency (EE%) was calculated with the equation defined as follows.
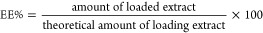
3The in vitro release profile of slime extracts was determined according to the Aker et al. study. Samples (*n* = 3) were incubated in 2 mL of 1× PBS (pH = 7.4) solution in an orbital shaker at 60 rpm and 37 °C. At specific time intervals, 200 μL of the 1× PBS was collected from each well and replaced with 200 μL of fresh 1× PBS to maintain a constant volume.^15^

### 2.5 Determination of Antimicrobial Activity

#### 2.5.1 Disc Diffusion Method

The antimicrobial activity of snail secretion extracts and in vitro release media of the nanofiber layer was evaluated with the disc diffusion method on Gram-positive *S. epidermidis* and Gram-negative *E. coli* bacteria, as described in our previous study.^20^ The nanofiber layer was incubated in 1× PBS solution at 37 °C for 24 h to obtain release media.

#### 2.5.2 Tube Dilution Method

The tube dilution method was carried out to assess the antimicrobial activity of snail secretion extracts against *S. epidermidis* and *E. coli* bacteria. Initially, the bacteria were cultured, and the turbidity was adjusted to McFarland 0.5. Subsequently, extract samples were prepared at concentrations of 1% M and 3% S for group 1 and 0.5% M + 0.5% S and 1.5% M + 1.5% S for group 2, which were then added to bacterial suspensions. These suspensions were inoculated onto Mueller–Hinton agar plates and incubated at 37 °C for 24 h. After the incubation period, bacterial colonies were counted and recorded to evaluate the antimicrobial effect of the mucus and slime secretions. All antimicrobial activity tests were carried out with three replicate sample groups.

### 2.6 In Vitro Cell Culture Studies

Human keratinocyte (HS2) and mouse fibroblast (NIH/3T3) cell lines were used for in vitro studies. High-glucose DMEM supplemented with 10% FBS, 1% l-glutamine, and 1% penicillin–streptomycin was used for cultivation media. HS2 and NIH/3T3 cells were cultivated on nanofiber layers and spongeous layers, respectively.

#### 2.6.1 Cytotoxicity Analysis of Bioactive Extracts

The in vitro cytotoxicity of mucus and slime extracts was evaluated according to the ISO-10993 standard with an indirect extraction method as previously described.^20^ Cell viability % was calculated with the equation defined as follows.
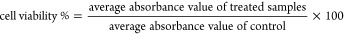
4

#### 2.6.2 In Vitro Wound Healing with the Scratch Assay

The in vitro scratch assay was performed according to Aker et al. research with the NIH/3T3 fibroblast cell line.^15^ Cells were incubated with 2 mM l-glutamine, 10% fetal bovine serum, 100 μg/mL streptomycin, and 100 U/mL penicillin in DMEM cultivation medium at 37 °C and 5% CO_2_. Experiments were carried out for 48 h in 96-well plates to observe the wound healing effect of mucus and slime extracts on cell migration and proliferation. A polylysine-coated surface was used to detect migration activity, whereas an uncoated polystyrene surface was used for proliferation capacity. An Olympus CX 31 was used to visualize and measure wound closure at each well for 0, 24, and 48 h periods, and the wound closure area was measured using Olympus DP2-BSW software. The wound closure % was calculated with the equation defined as follows.
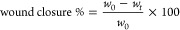
5Here, *w*_0_ is the initial wound width and *w_t_* is the wound width at the incubation period.

#### 2.6.3 Cell Proliferation of Bilayer Wound Dressing

HS2 and NIH/3T3 cells were seeded on spongeous and nanofiber layers of scaffolds (*n* = 3). Cell proliferation on samples was investigated for 14 days, and the samples were incubated at 37 °C and 5% CO_2_ during the cultivation period. Before cell seeding, the scaffolds were sterilized with 70% (v/v) ethanol solution for 24 h and then washed with 1× phosphate-buffered saline (PBS) solution 3 times. The samples were conditioned with cell culture media for 2 h, and then HS2 and NIH/3T3 cells were seeded on upper and bottom layers at a density of 1 × 10^6^ cells/mL, respectively. After cell seeding, the samples were incubated at 37 °C and 5% CO_2_ for 4 h to provide cell attachment. DMEM high glucose (10% FBS, 1% l-glutamine, and 1% penicillin–streptomycin) was used as the cultivation medium of HS2 and NIH/3T3 cell lines. The culture medium was changed twice a week. The WST-1 cell viability assay kit (Biovision) was used to determine cell viability (*n* = 3) at 440 nm (Varioskan Flash, Thermo Fisher Scientific). Cell proliferation was given as the absorbance change with incubation periods.

#### 2.6.4 Cell Attachment and Spreading

HS2 and NIH/3T3 cells were seeded into a porous layer and a nanofiber layer and cultured for 3 days to observe cell attachment and spreading. Samples were fixed with 4% paraformaldehyde (v/v) (PFA) solution and then washed three times with 1× PBS solution to remove PFA. After the fixation protocol, the cell membrane was permeabilized with 0.1% Triton X-100 for 5 min, and Alexa Fluor 488 and DAPI staining protocols were carried out. Fluorescence microscopy (ZEISS Observer Z1) was used to observe the cell cytoskeleton and nuclei on the bilayer scaffold surface.

#### 2.6.5 Hydroxyproline (HP) Assay

HP secretion of HS2 cells on the nanofiber layer (*n* = 3) was evaluated using a colorimetric HP assay kit according to the manufacturer’s protocol. Total HP content was measured at 14 days for the HS2 cell line at 550 nm.

#### 2.6.6 Type I Collagen Content

Type I collagen secretion of NIH/3T3 cells on the spongeous layer (*n* = 3) was investigated with the Sandwich ELISA assay kit according to the manufacturer’s protocol. Type I collagen content was spectrophotometrically measured at 450 nm on days 7 and 14 of incubation.

#### 2.6.7 Glycosaminoglycan (GAG) Content

Glycosaminoglycan production of NIH/3T3 fibroblasts on the spongeous layer was investigated with a spectrophotometric proteoglycan detection kit on 7 days of incubation. First, samples were exposed to papain extraction by incubating in papain solution (papain, dithiothreitol (DTT), ethylenediamine tetraacetic acid (EDTA)) for 6 h at 60 °C. Then, GAG content was determined spectrophotometrically at 530 nm using dimethylmethylene blue DMMB solution.

#### 2.6.8 Safranin-O Staining

GAG secretion of NIH/3T3 cells on the spongeous layer was visualized using Safranin-O staining after 14 days of incubation. The samples were washed with 1× PBS solution, and cell fixation was carried out by incubating in 4% (PFA) solution. Afterward, bilayer wound dressings were stained with Safranin-O for 10 min and then washed with 95% ethanol solution. Finally, the samples were washed with 1× PBS solution and visualized with an optical microscope.^28^

### 2.7 Statistical Analysis

In this study, the statistical differences between all groups were analyzed using one-way and two-way ANOVA with Tukey’s multiple comparison test statistical methods (*p* < 0.05). Three samples were used in characterization tests and bioactivity tests. Five samples were used for mechanical analysis. The experimental data were given with the standard error of the mean.

## 3 Results and Discussion

### 3.1 Morphology and Structure of Bilayer Wound Dressing

**Figure 2:**
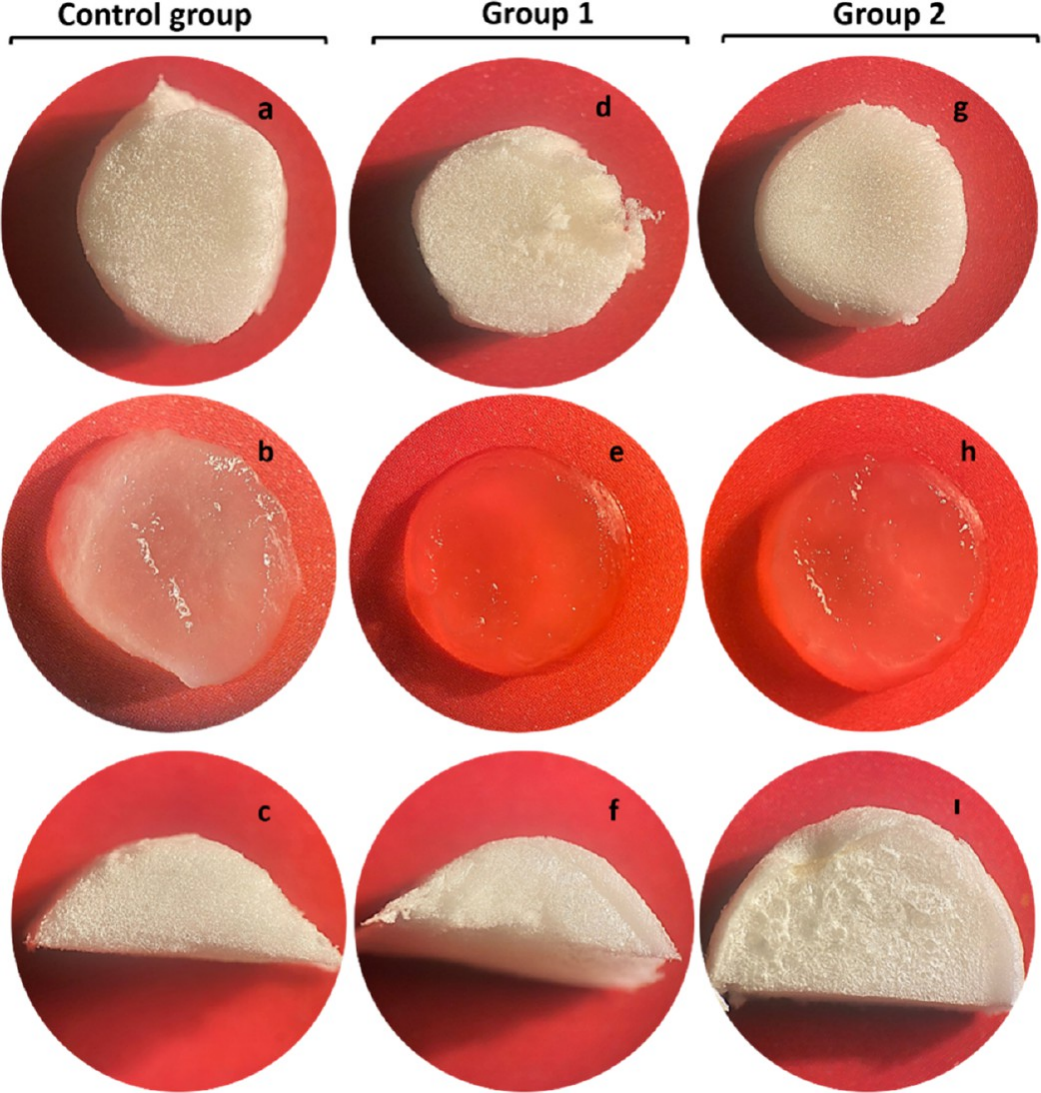
Stereoimages (2× magnifications) of bilayer wound dressings in dry (a, d, g) and wet (b, e, h) conditions. (c, f, i) Images indicated a cross-sectional view of the samples (sample size: 13 mm diameter and 10 mm height).

The structural integrity and stability of bilayer wound dressings were investigated by using stereomicroscopy. Stereoimages revealed that the nanofiber layer (upper layer) was physically integrated within the spongeous layer (bottom layer) under both dry and wet conditions (Figure 2). Cross-sectional images of the bilayer wound dressings further demonstrated good compatibility between the nanofiber and spongeous layers., Each layer was fabricated by using a chitosan matrix. Hence, high polymer–polymer interaction was obtained within the layers.

**Figure 3:**
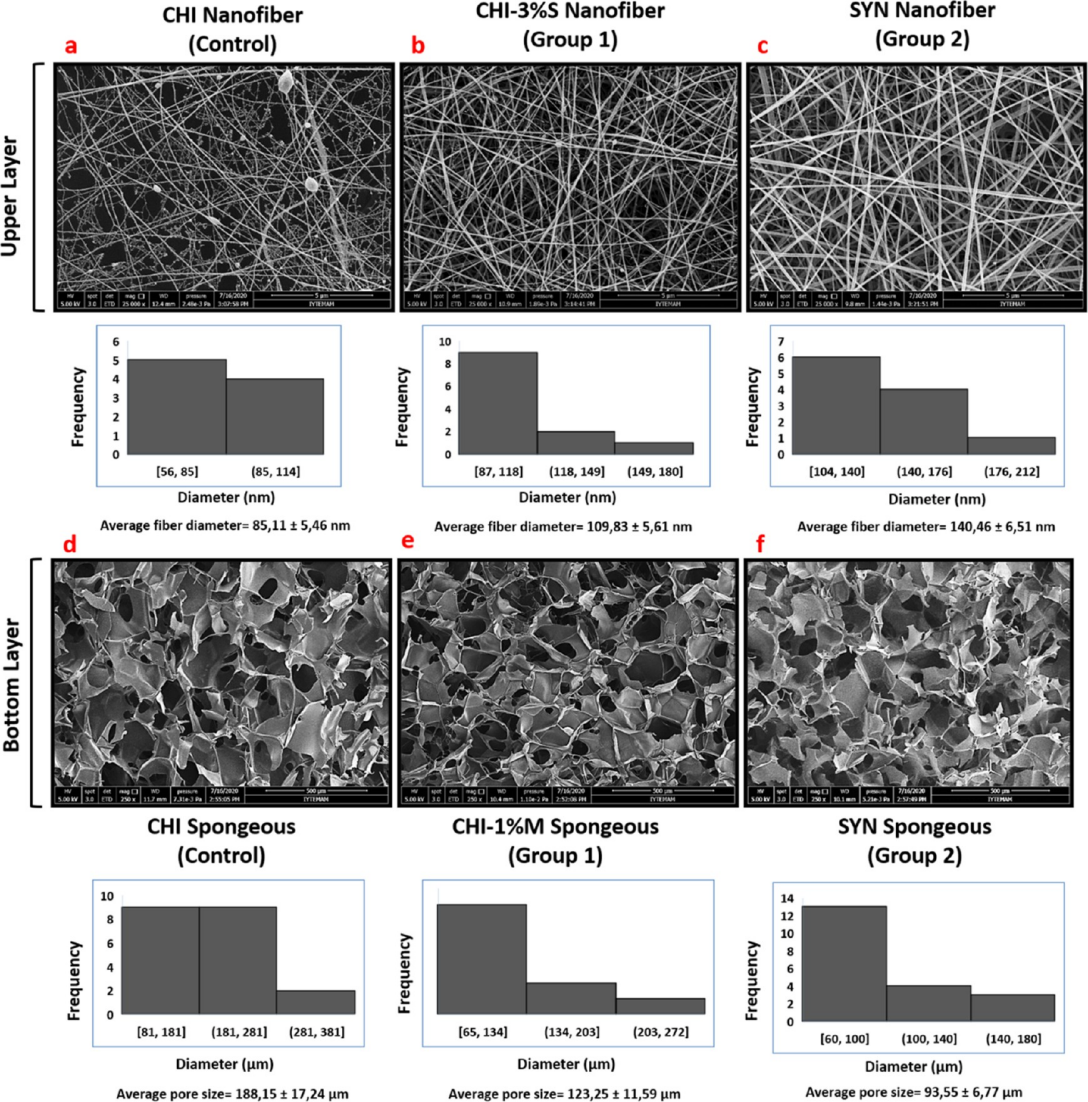
SEM images and histogram data of the control group, group 1, and group 2. Images were given as (a, d), (b, e), and (c, f), respectively (magnification of nanofiber layers is 25,000× and magnification of spongeous layers is 250×; scale bars for nanofiber and spongeous groups are 5 and 500 μm, respectively).

**Figure 4:**
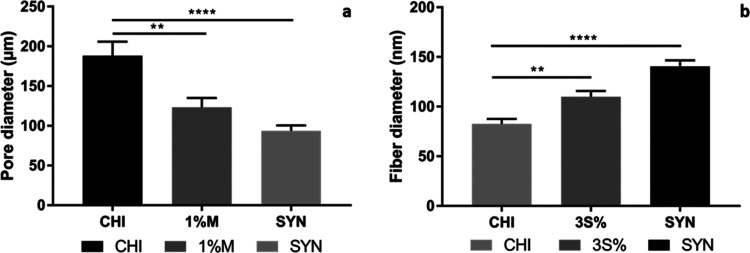
(a) Pore diameter of the spongeous layer and (b) fiber diameter of the nanofiber layer for bilayer wound dressings (*p* < 0.01 is represented as **; *p* < 0.0001 is represented as ****).

The morphology of bilayer wound dressings was examined through SEM analysis, focusing on both porous and nanofiber layers individually. Our findings revealed that the upper layer exhibited a nanofiber structure, while the lower layer displayed a microporous morphology (Figure 3). Using ImageJ software, we measured the average pore sizes and nanofiber diameters for different groups: CHI, CHI–1%M, and SYN. The results showed that porous bottom layers had average pore sizes of 188.15 ± 17.24, 123.25 ± 11.59, and 93.55 ± 11.59 μm, while average nanofiber diameters were measured as 85.11 ± 5.46, 109.83 ± 5.61, and 140.46 ± 6.51 nm for the upper layers of CHI, CHI–3S, and SYN groups, respectively (Figure 4). SEM images indicated a homogeneous distribution of pores in the porous layers, with an increase in the surface area of the pore walls and a reduction in pore sizes when the extracts were incorporated into the chitosan matrix. In addition, an increase in nanofiber diameters was observed for groups 1 and 2, along with a more uniform distribution of nanofibers compared to the CHI control group. Histogram data also showed that the extract loading increased the average diameter of the nanofiber layer in the ranges of 87–118 and 104–140 nm, while it decreased the sponge layer in the ranges of 65–134 and 60–100 μm, respectively.

The minimum pore size required for attachment and proliferation of fibroblast and keratinocyte cells for a scaffold is 5–15 μm, which is critical for establishing an adequate microenvironment and ensuring efficient nutrient transport. Additionally, the optimal pore size range for skin regeneration is between 20 and 125 μm. These results suggest that the obtained average pore size and nanofiber diameter ranges for bilayer samples align with previous studies and hold promise for skin tissue regeneration.^29,30^

**Figure 5:**
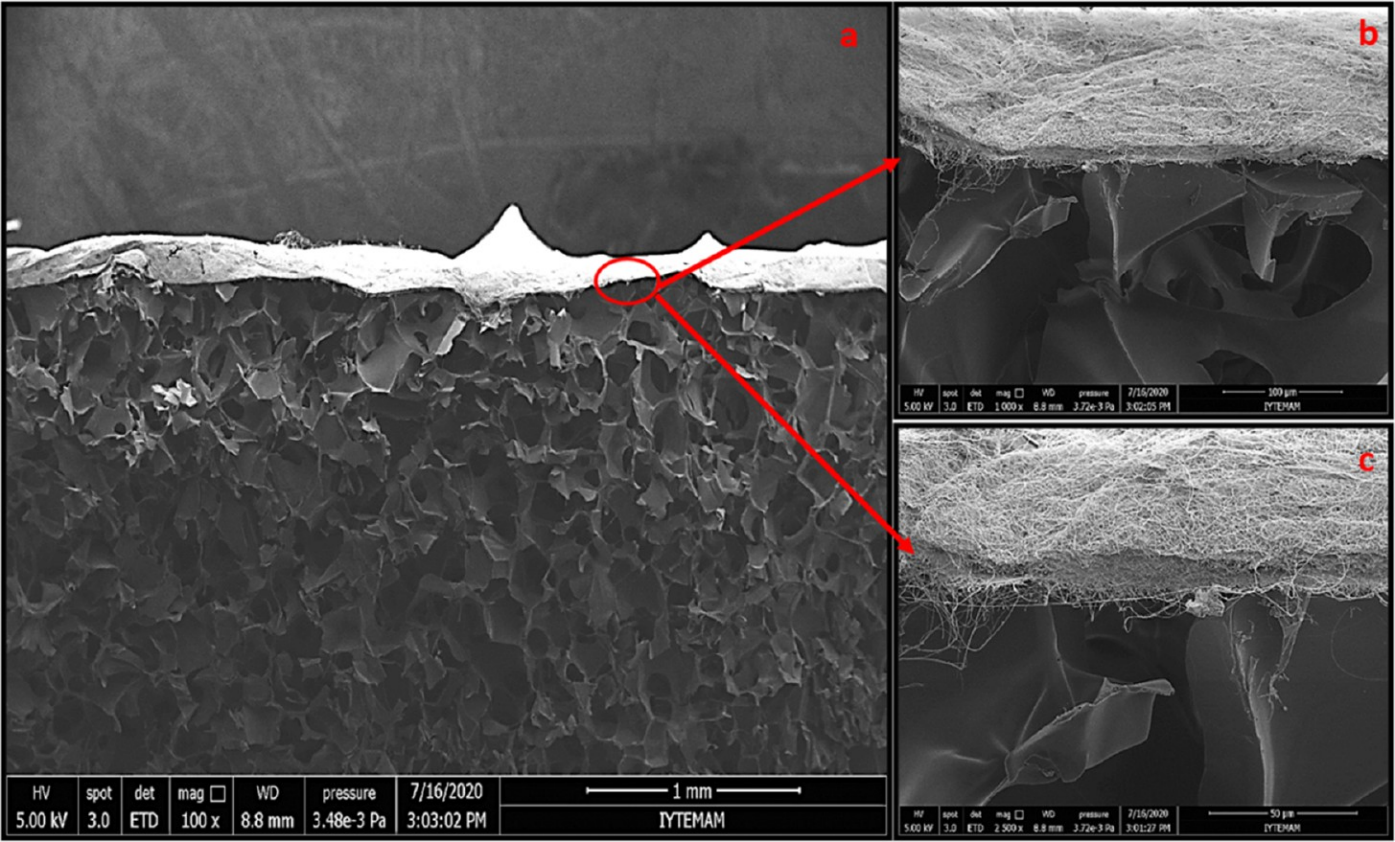
SEM images of a cross section for the control group. Scale bars: (a) 1 mm, (b) 100 μm, and (c) 50 μm.

**Figure 6:**
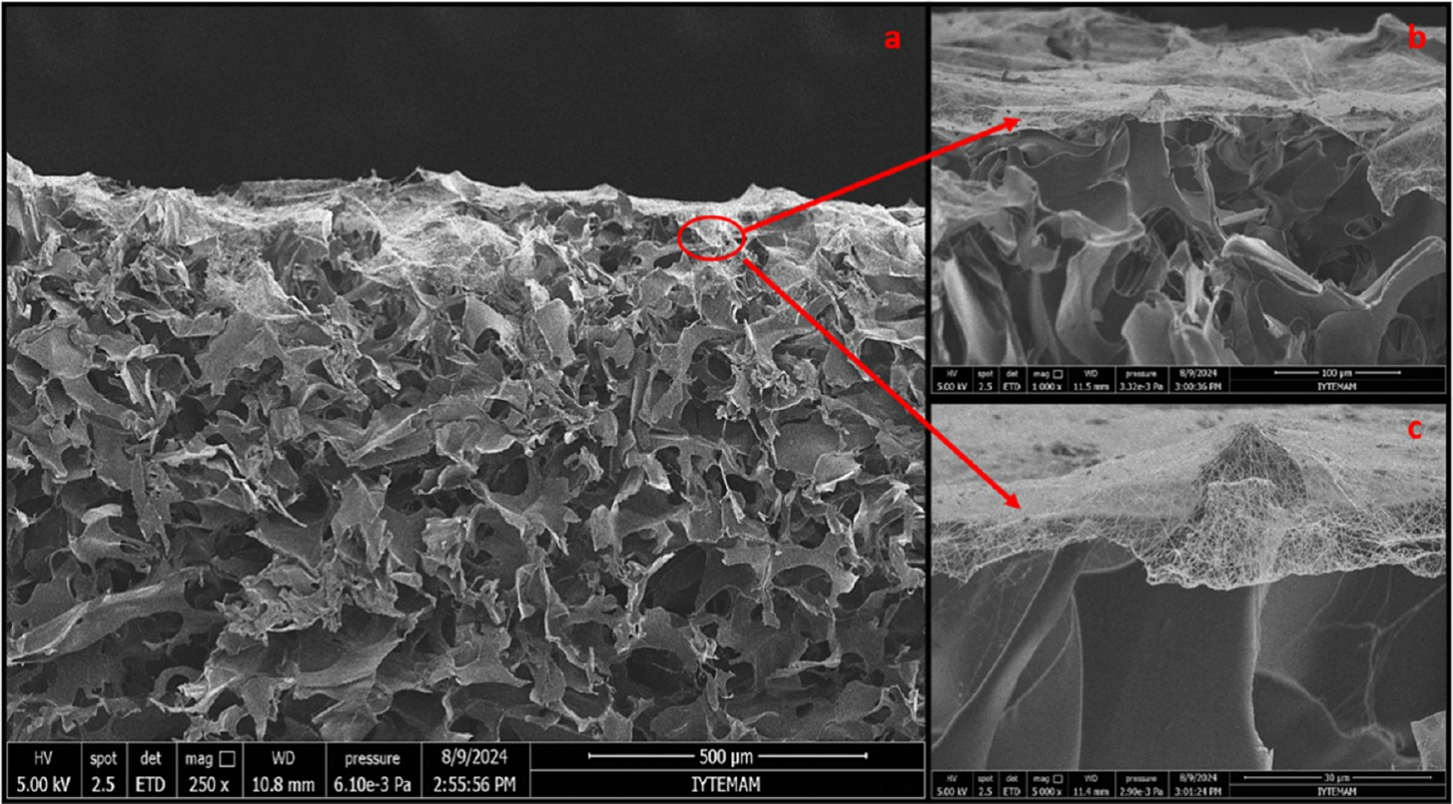
SEM images of a cross section for group 1. Scale bars; (a) 500 μm, (b) 100 μm, and (c) 30 μm.

**Figure 7:**
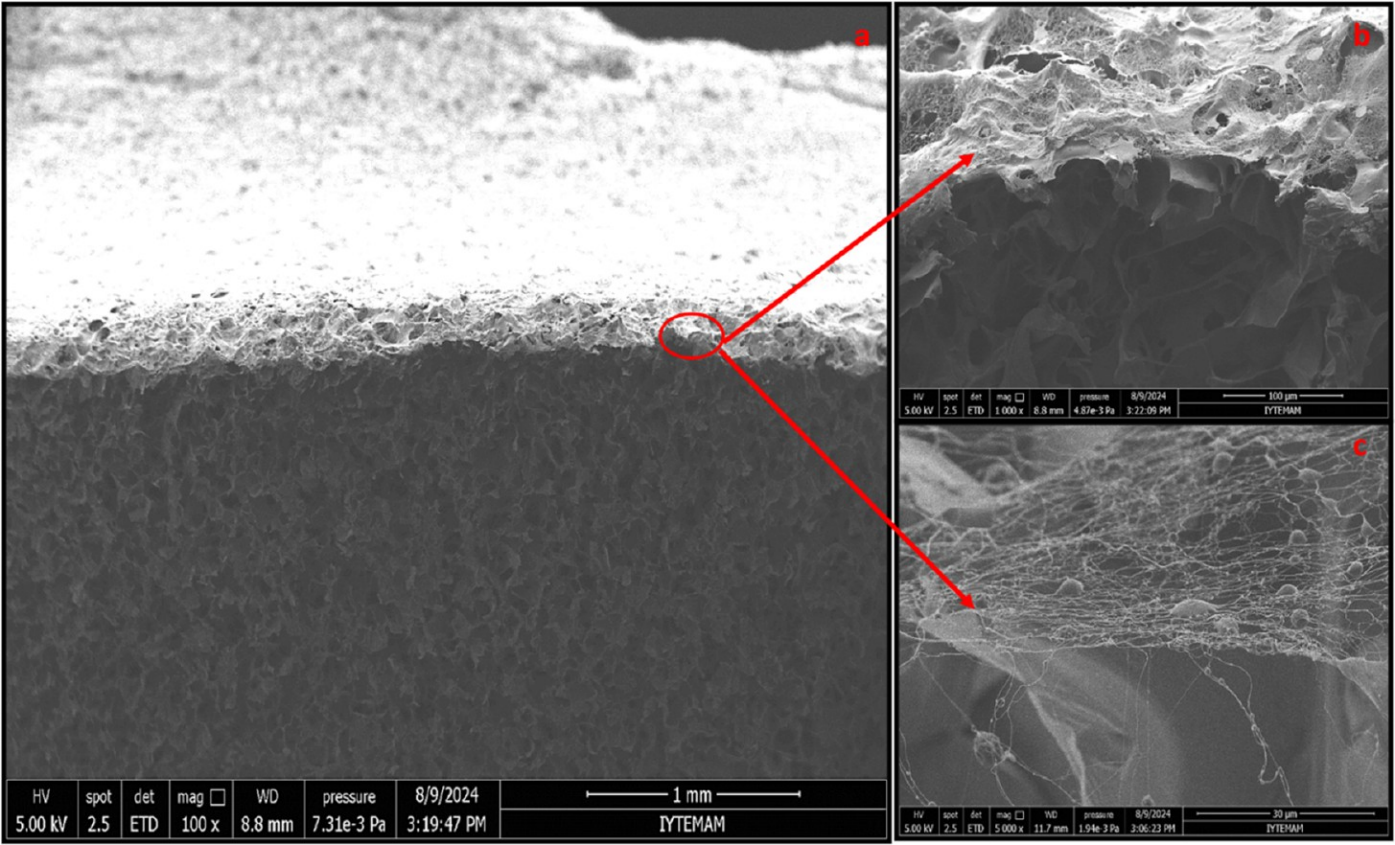
SEM images of a cross section for group 2. Scale bars: (a) 1 mm, (b) 100 μm, and (c) 30 μm.

All groups were coated with nanofibers under similar conditions (1 mL volume of solutions), and the thicknesses of spongeous and nanofiber layers of bilayer wound dressings are shown in Figures 5,6, and 7. In our study, the thicknesses of the porous and nanofiber layers were calculated for the control group as 1.8 ± 0.39 mm and 8.8 ± 0.34 μm, for group 1 as 1.58 ± 0.01 mm and 5.93 ± 0.7 μm, and for group 2 as 1.50 ± 0.01 mm and 6.71 ± 0.4 μm, respectively. In the literature, the thicknesses of the dermis and epidermis layers were reported as 0.3–3 mm and 0.05–1.55 mm, respectively.^31^ The thickness range of the designed porous bottom layer was found to be consistent with the thickness range of the dermis in the literature.

### 3.2 Open Porosity of Spongeous Layers

Porosity is an essential parameter in tissue engineering constructs designed to promote effective wound healing. Highly porous tissue scaffolds enhance cell adhesion and proliferation, facilitate the transfer of nutrients and oxygen, and contribute to mechanical strength, thus accelerating the wound healing process within the tissue.^32^ In our study, the open porosity of the bottom layers was assessed utilizing the liquid displacement method with the corresponding porosity percentage data presented in Table 2. The findings revealed that the incorporation of mucus and slime extracts led to an augmentation in porosity percentage, surpassing 75% for all groups, and contributed to improved microstructure homogeneity. Notably, in open porosity measurements, no statistically significant difference was observed among the groups. Previous research has established that the optimal porosity percentage for wound dressings should ideally be within the range of 60–90%.^33^ Thus, the open porosity of fabricated bilayer wound dressings was found in the range and favorable to promote skin tissue regeneration.

**Table 2:** Open Porosity % of Spongeous Layers

scaffold groups	open porosity %
CHI	77.3 ± 2.47
CHI–1% M	82 ± 4.38
SYN	81.3 ± 2.85

### 3.3 Water Absorption of Bilayer Wound Dressings

**Figure 8:**
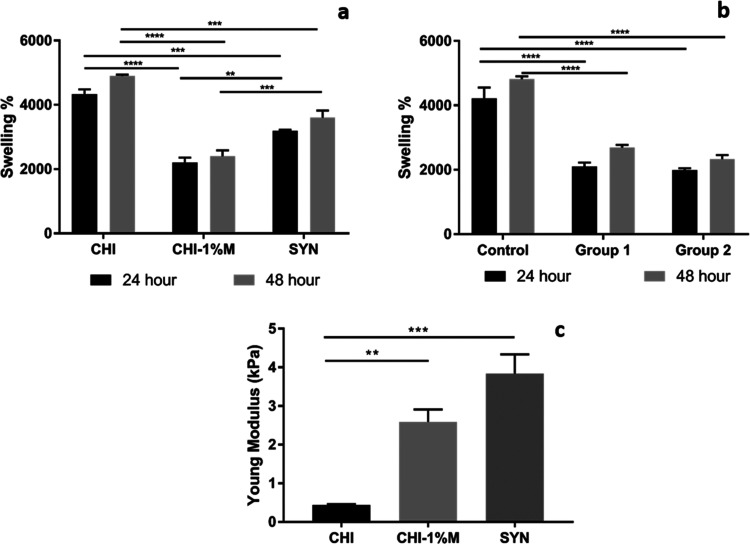
Swelling percentages of (a) spongeous layer and (b) bilayer wound dressings for 24 and 48 h. (c) Young modulus of spongeous layer wound dressings (*p* < 0.01 is represented as **; *p* < 0.001 is represented as ***; *p* < 0.0001 is represented as ****).

Water absorption characteristics of the porous layer and bilayer wound dressing groups were evaluated with swellings tests. Samples were immersed in 1× PBS solution, and then the weight changes of the samples were measured at 24 and 48 h (Figure 8a,b). Results showed that the water uptake capacity of all porous and bilayer groups showed an increasing trend up to an incubation period of 48 h. However, mucus and mucus–slime extract loading decreased the swelling capacity of the chitosan matrix for both single and bilayer samples. Although mucus and slime extract incorporation decreased the water absorption capacities of single-layer and bilayer wound dressings, the samples still have a high % swelling ratio in the range of 2300–4050. Similarly, Angulo et al. demonstrated that the swelling % of mucus-loaded scaffolds decreased with increasing mucus concentration.^34^

### 3.4 Mechanical Properties of Spongeous Layers

An effective scaffold should serve as a barrier against infection and external damage while exhibiting suitable mechanical strength.^35^ Mechanical properties are fundamental in determining the load-bearing capacity of scaffolds that exhibit adequate durability and facilitate the healing of soft tissues beneath the skin by providing a suitable environment for attachment and proliferation of cells such as fibroblasts and keratinocytes while preventing damage under load.^3,36^ In addition, the adequacy of the mechanical properties is crucial for the integration of scaffolds with surrounding tissues. Effective integration promotes optimal blood flow (vascularization), thereby enhancing the speed of wound healing.^37^

The mechanical properties of the spongeous layers were examined with a compression test to measure Young’s modulus (Figure 8c). The findings revealed that the incorporation of mucus and slime extracts into the chitosan matrix enhanced Young’s modulus. The highest modulus was recorded for the SYN group as 4.86 kPa, while the CHI–1% M group exhibited a modulus of 3.23 kPa. This increase may arise from morphological alterations with extract incorporation in the chitosan matrix. Mucus and slime extracts altered the microstructure with an increase in the surface area of the pore walls and a decrease in the pore sizes. In addition, the rich mineral content of *H. aspersa* snail extract mainly composed of Ca, Cl, K, P, and Mg may enhance the mechanical strength by incorporating in a polymer matrix.^38^ Notably, a study by Angulo et al. demonstrated similar results, where gelatin/chitosan scaffolds incorporated with snail mucus and aloe vera extract exhibited an increase in Young’s modulus.^34^

### 3.5 Protein Adsorption

**Figure 9:**
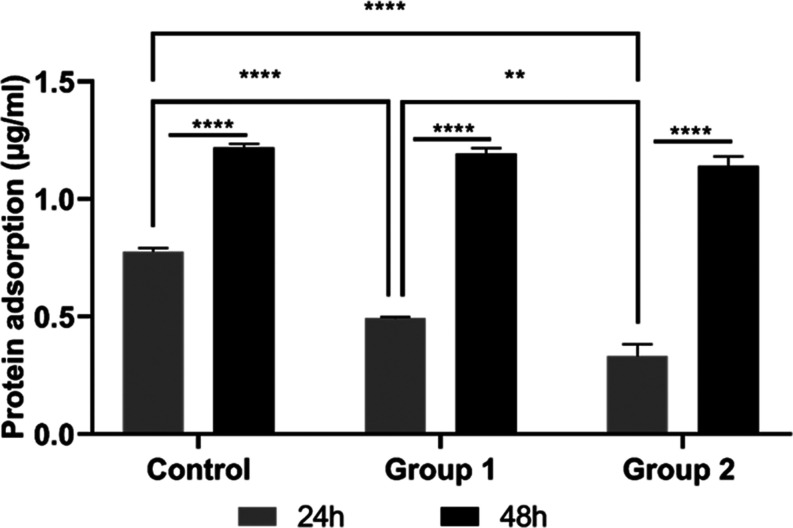
Protein adsorption of the control group, group 1, and group 2 bilayer wound dressings at 24 and 48 h of incubation (*p* < 0.01 is represented as **; *p* < 0.0001 is represented as ****).

Protein adsorption from body fluids represents one of the initial interactions at the tissue–material interface.^39^ As such, the ability of implantable biomaterials to adsorb proteins is a crucial factor for avoiding recognition as a foreign body reaction, facilitating cell attachment, differentiation, and wound healing on material surfaces.^40^ In our study, we observed a decrease in protein adsorption for groups 1 and 2 compared to the control group at 24 h of incubation. Protein adsorption differences between groups were found statistically significant at 24 h. However, all groups exhibited an increase in protein adsorption capacity up to 48 h, and this increasing trend by incubation time was found statistically significant (Figure 9). Groups 1 and 2 demonstrated a notable increasing trend from 0.5 to 1.19 and 0.33 to 1.8 μg/mL, respectively, from 24 to 48 h, whereas the control group showed an increase from 0.8 to 1.32 μg/mL in protein adsorption capacity. The mucoadhesive characteristics of both mucus and slime extracts^41^ may lead to an increase in the protein adsorption trend from 24 to 48 h of incubation. Results indicated that protein adsorption increased 1.5-fold on the chitosan control group and 2.4-fold on group 1, whereas adsorption increased 4.5-fold on group 2, showing the positive synergic effect of mucus and slime extracts.

### 3.6 In Vitro Release Study

**Figure 10:**
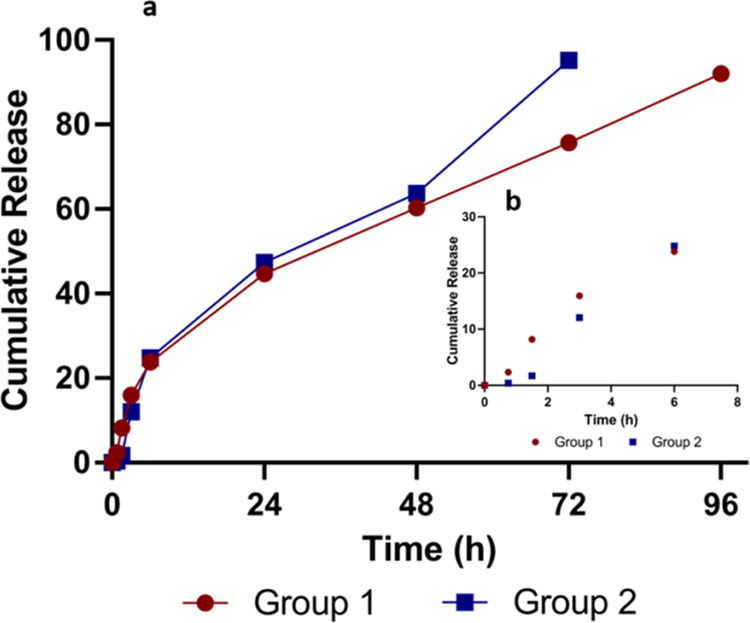
Cumulative release (a) and burst release (b) of slime extract from nanofiber layers of groups 1 and 2.

Nanofiber layers fabricated with the electrospinning method were recently used to encapsulate bioactive and antimicrobial agents to obtain a controlled release with high encapsulation efficiency due to their higher surface area.^42^ In this study, antimicrobial slime extract was loaded in a chitosan matrix with an electrospinning method to obtain an antibacterial barrier nanofiber layer with a controlled release mechanism while mimicking the epidermis via inducing keratinocyte activity. In vitro release of antimicrobial slime extract in the genipin cross-linked chitosan nanofiber matrix was investigated to evaluate the release rate and mechanism. The encapsulation efficiency of slime extracts on nanofiber layers was determined by the acetic acid hydrolysis method given in Section 2.4.6. In group 1, slime extract loading was carried out with 70% encapsulation efficiency, whereas slime extract was loaded in the group 2 nanofiber layer with 83% encapsulation efficiency. In the group 2 nanofiber layer, higher encapsulation efficiency was obtained due to the possible synergistic effect of mucus and slime extract in the polymer matrix. The in vitro release study was spectrophotometrically determined at 280 nm, which is the main UV–vis peak of slime extract. In the literature, snail extract was found to exhibit maximum absorption at 293 nm with UV–vis spectroscopy.^43^ Flippo and co-workers also indicated that the specific UV–vis peaks of *H. aspersa* snail extracts between 250 and 300 nm are related to the structural components such as amino acids with aromatic groups that form antibacterial peptides in snail composition.^44^ Release profiles showed that a burst release of slime extract was obtained up to 6 h, then the release rate decreased, and a sustained release region was obtained up to 3–4 days (Figure 10). Results showed that, initially, 23% of slime extract was released in the group 1 nanofiber layer, whereas 24% of slime extract was released from group 2 up to 6 h. After the initial release, group 1 released 92% of the slime extract with a sustained profile after an incubation period of 96 h. However, group 2 released 95% of the slime extract after 72 h of incubation. Both nanofiber layer groups exhibited similar release profiles up to 48 h with releasing 60–63% of slime extract. After 48 h, the release rate of group 2 increased when compared with group 1. This may arise from the possible dissolution of slime extract with a higher concentration (3%) loaded on the surface of the nanofiber. The burst release of slime extract up to 6 h of incubation can block the initial bacterial attack on the wound site, and the sustained release of the extract can eliminate microbial contamination during the wound healing period.

As in the conventional electrospinning method, a bioactive agent can be mixed with a polymer solution before nanofiber production. In this system, drug release from nanofibers is carried out via diffusion or polymer degradation via erosion. Therefore, the rate of bioactive agent release from nanofibers is generally controlled by dissolution and/or diffusion mechanisms.^42^ To investigate the release kinetics and the mechanism of slime extract release from nanofiber layers of the bilayer wound dressing groups, kinetic models were applied to release data. Results indicated that slime extract release from group 1 and group 2 nanofiber layers fit the first-order and Higuchi models with high correlation coefficient values (*R*^2^), as given in Table 3. The first-order model explains the mechanism of the burst release region as the thin nanofiber layer may release the slime extract from the polymer surface up to 6 h of incubation. However, at the sustained release region, the Higuchi model explains the diffusion mechanism of slime extract encapsulated in a chitosan nanofiber matrix chemically cross-linked with genipin.

**Table 3:** Release Kinetic Coefficients for Nanofiber Layers

kinetic models (*R*^2^)	group 1	group 2
first-order	0.9893	0.9969
Higuchi	0.9998	0.9773

### 3.7 Antimicrobial Activity of Snail Extracts and Bilayer Wound Dressing

**Figure 11:**
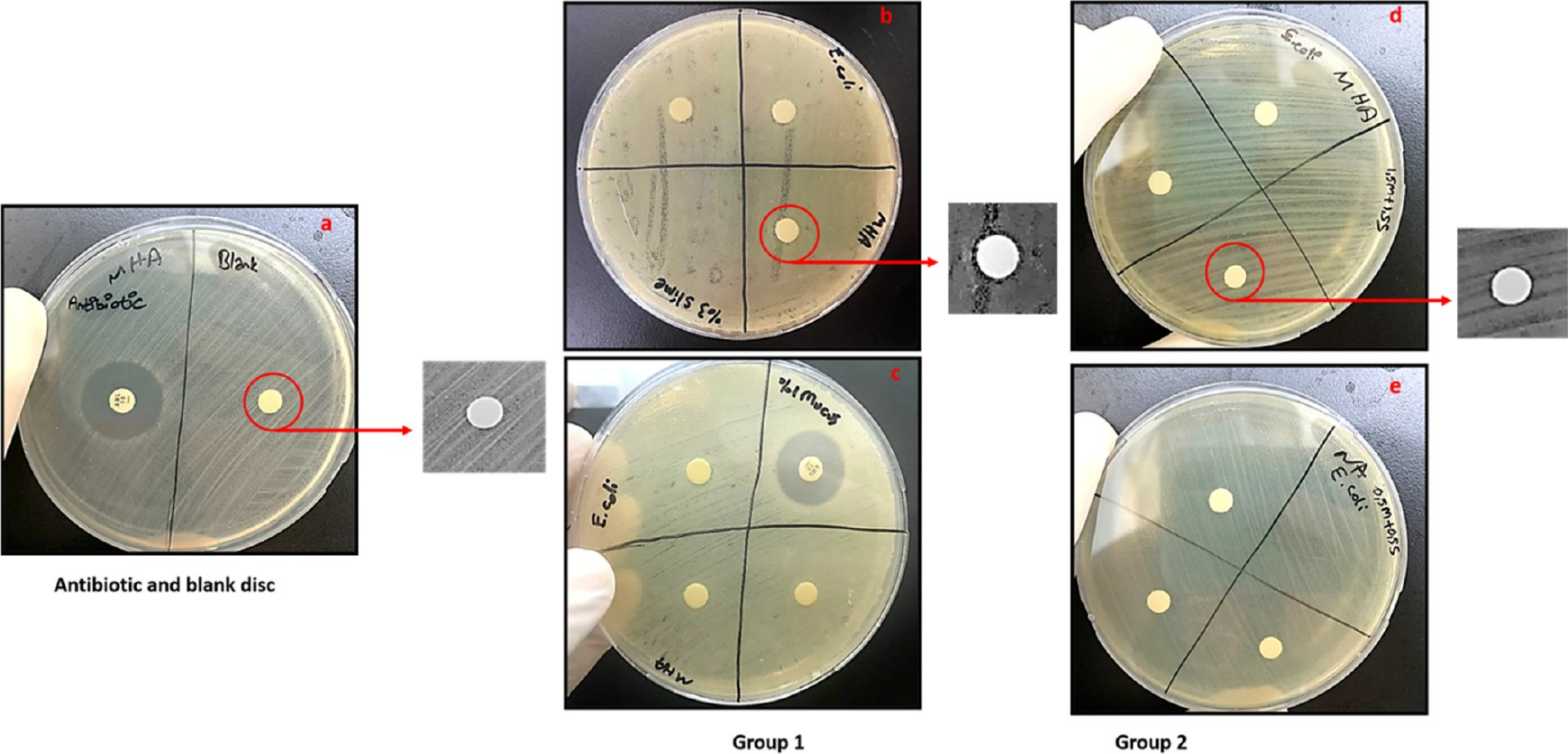
Disc diffusion zones of (a) antibiotic and blank, (b) 3% slime, (c) 1% mucus, (d) 1.5% mucus + 1.5% slime, and (e) 0.5% mucus + 0.5% slime extract solutions against *E. coli* bacteria.

**Figure 12:**
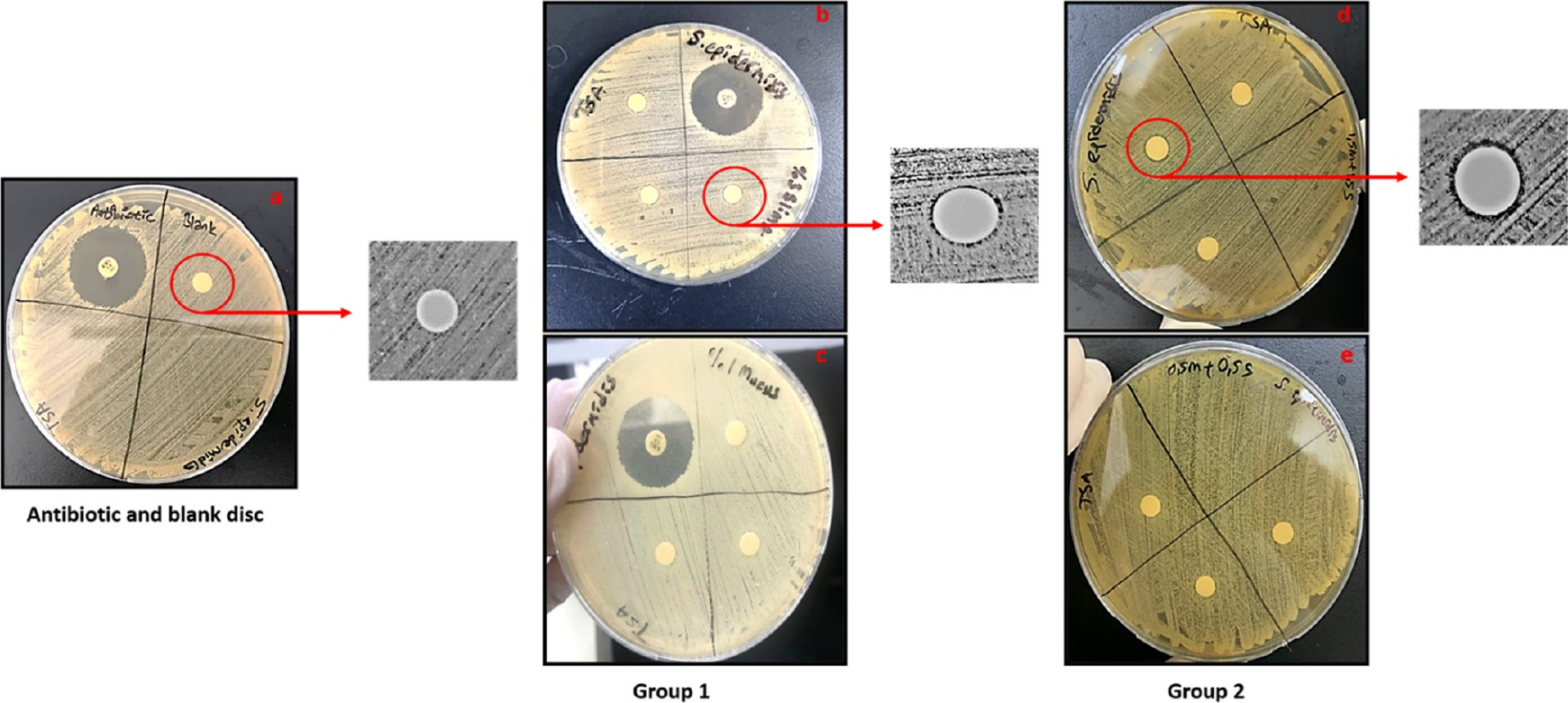
Disc diffusion zones of (a) antibiotic and blank, (b) 3% slime, (c) 1% mucus, (d) 1.5% mucus + 1.5% slime, and (e) 0.5% mucus + 0.5% slime extract solutions against *S. epidermidis* bacteria.

**Figure 13:**
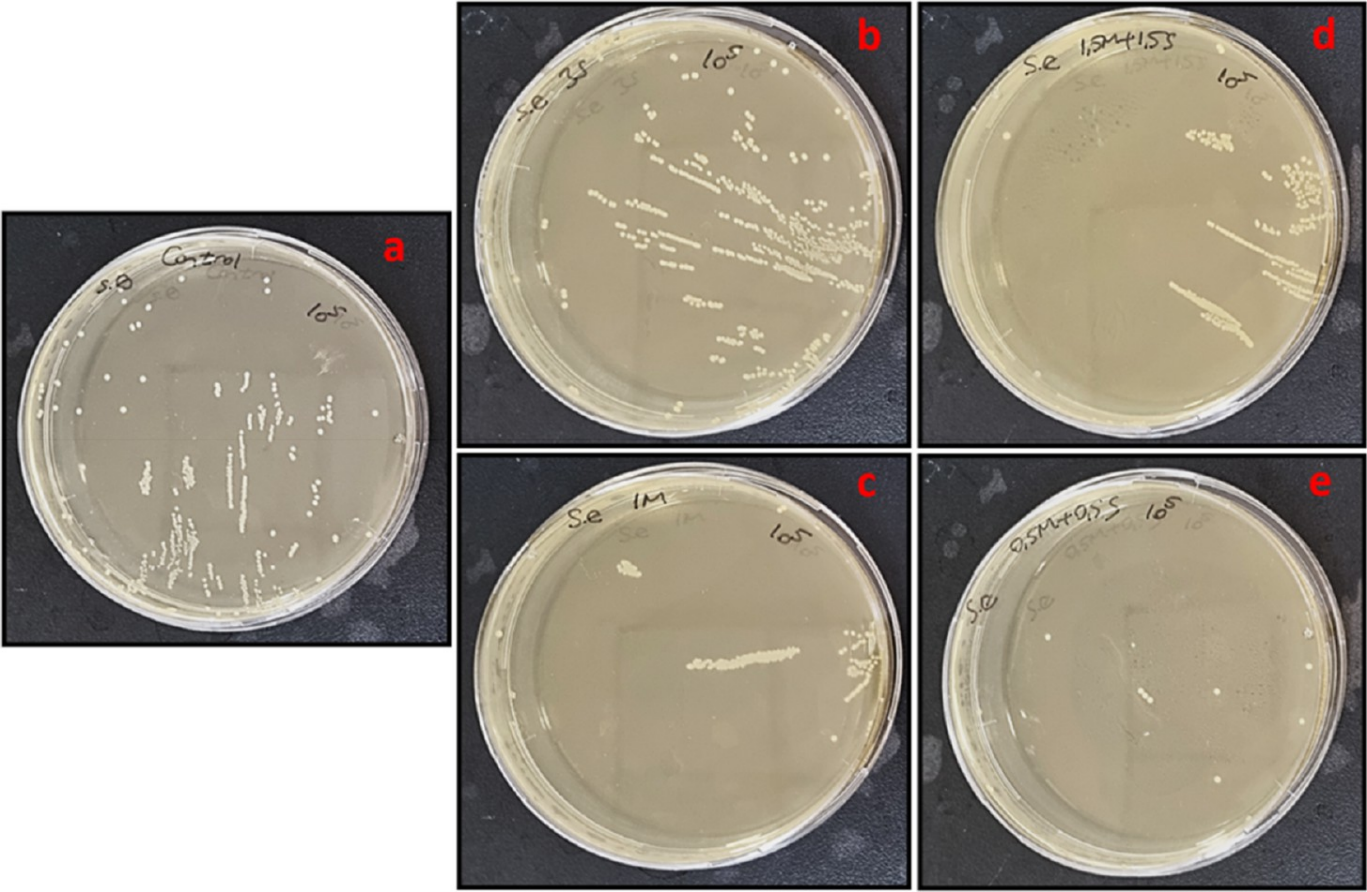
Tube dilution results of (a) control, (b) 3% slime, (c) 1% mucus, (d) 1.5% mucus + 1.5% slime, and (e) 0.5% mucus + 0.5% slime extract solutions against *S. epidermidis* bacteria colonies at 24 h of incubation.

**Figure 14:**
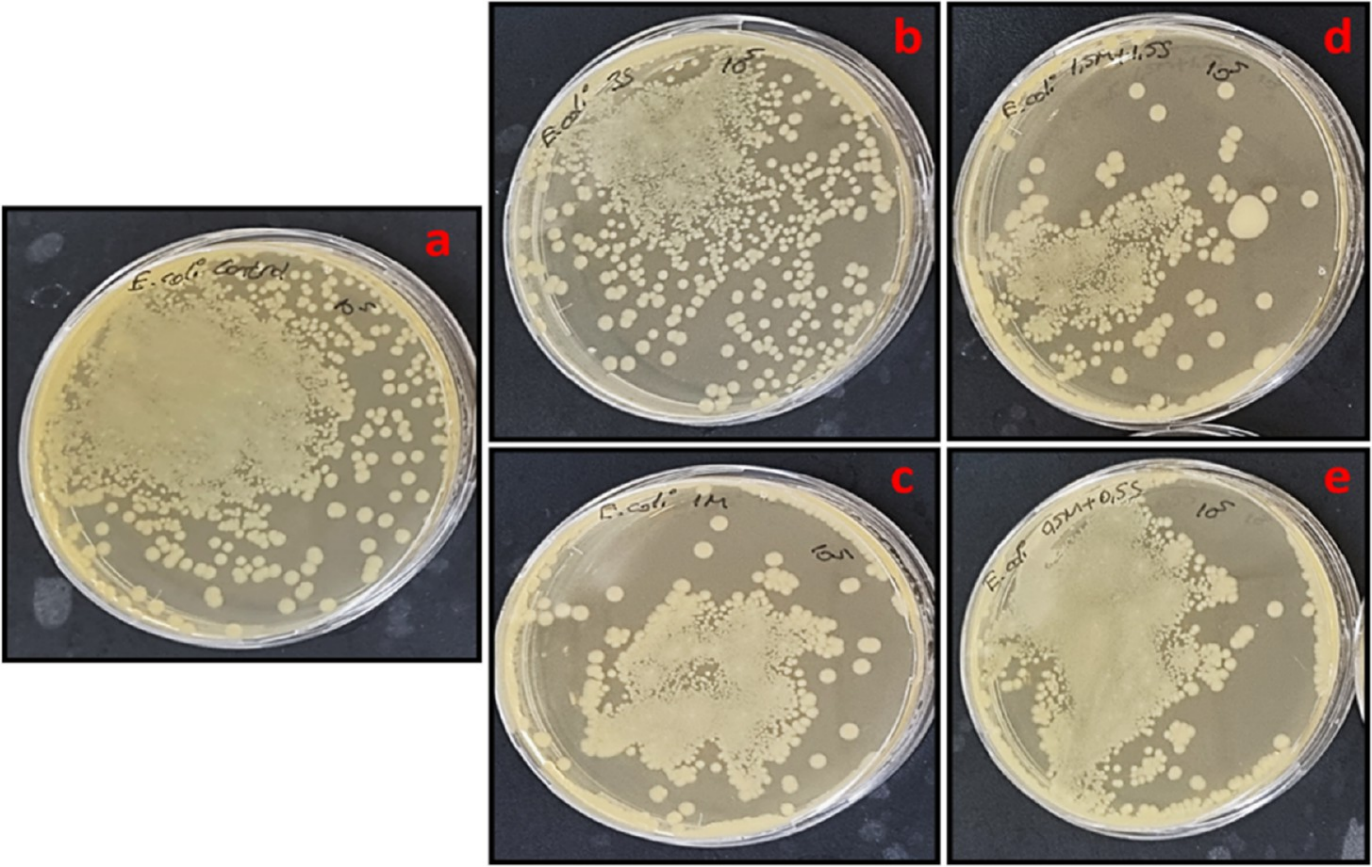
Tube dilution results of (a) control, (b) 3% slime, (c) 1% mucus, (d) 1.5% mucus + 1.5% slime, (e) 0.5% mucus+0.5% slime extract solutions against *E. coli* bacteria colonies at 24 h of incubation.

In this study, we investigated the antimicrobial activity of mucus and slime extracts using the disc diffusion and tube dilution methods against the Gram-positive *S. epidermidis* and Gram-negative *E. coli* bacteria. Mucus and slime solutions were prepared at concentrations of nanofiber layers of 3% S for group 1 and 1.5% M + 1.5% for group 2. Disc diffusion results indicated that extract solutions showed small inhibition zones for both *S. epidermidis* and *E. coli*. For *S. epidermidis*, clear zones of inhibition were observed at 3% S and 1.5% M + 1.5% S concentrations around the discs, whereas no distinct zone was observed for *E. coli* (Figures 11 and 12). Zone diameters are depicted in Table 4. These results may arise from the mucoadhesive properties of extracts, which may lead to the insufficient release of solutions from discs to the agar surface. Thus, the tube dilution method was carried out for concentrations used in nanofiber layer groups (3% S and 1.5% M–1.5% S). After 24 h of incubation, bacterial colony counting was carried out, and the results are depicted in Table 5. According to the results, mucus and slime extracts reduced the bacterial colonies slightly compared to the control group (Figures 13 and 14). Both mucus, slime, and synergic groups showed distinct antimicrobial effects against *E. coli* by significantly decreasing the colony number from 10^10^ to 10^8^. However, all extract groups showed mild antimicrobial effects against *S. epidermidis*. The colony number decreased from 4.8 × 10^8^ to 1.19 × 10^8^ for the 1% M group, while synergic groups (0.5% M–0.5%S; 1.5% M–1.5% S) showed a higher antimicrobial effect by decreasing the colony number to 1.2 × 10^7^ and 2.38 × 10^8^, respectively.

**Table 4:** Inhibition Zone Diameters of Mucus and Slime Extract Solutions

groups	inhibition zones of *S. epidermidis* (mm)	inhibition zones of *E. coli* (mm)
amoxicillin	10	10
1% mucus		
3% slime	1 ± 0	
0.5% mucus + 0.5% slime		
1.5% mucus + 1.5% slime	1 ± 0	

**Table 5:** Bacterial Colony Counts Were Quantified Using the Tube Dilution Method

groups	*S. epidermidis* (cfu/mL)	*E. coli* (cfu/mL)
control	4.8 × 10^8^	5.2 × 10^10^
3% S	4.45 × 10^8^	7.2 × 10^8^
1.5% M + 1.5% S	2.38 × 10^8^	9.18 × 10^8^

**Figure 15:**
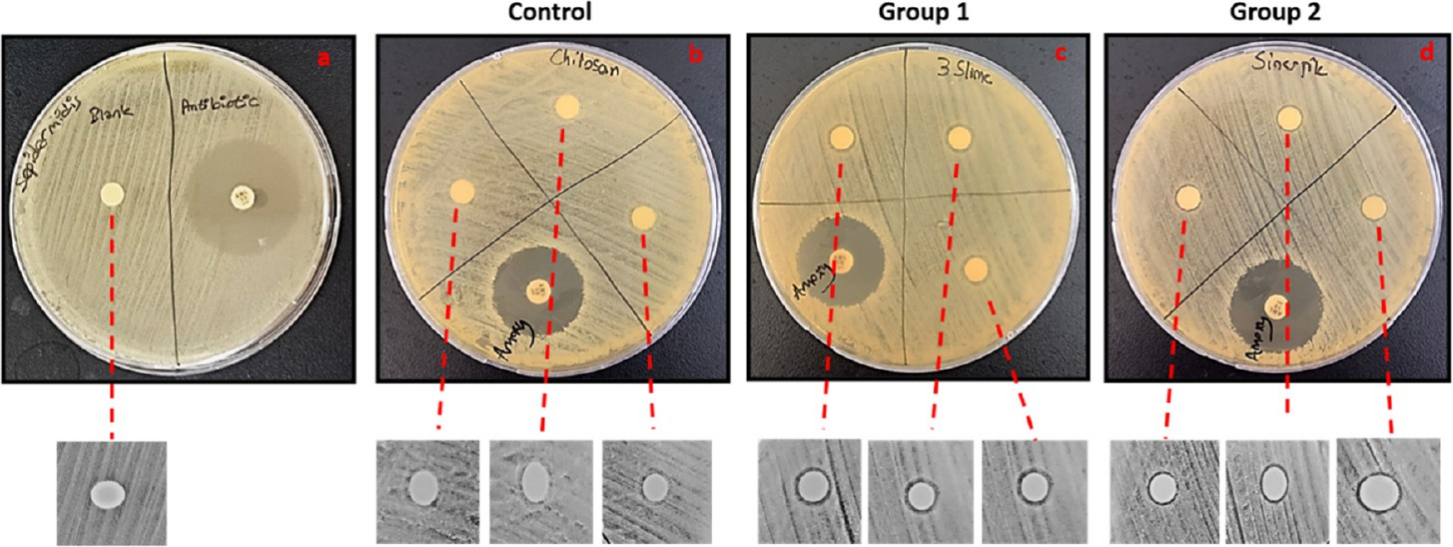
Disc diffusion zones of release media of (a) blank and antibiotic disc, (b) control group, (c) group 1, and (d) group 2 against *S. epidermidis* bacteria.

Additionally, antimicrobial experiments were conducted for the release solutions of the nanofiber layer at 24 h (Figure 15). The wound dressings were incubated in 1× PBS solution at 37 °C for 24 h, and the release solutions were tested using disc diffusion methods to assess the antimicrobial activity of the soluble bioactive content of mucus and slime extracts against *S. epidermidis* bacteria. The results indicated that the wound dressings released mucus and slime, exhibiting slight antimicrobial activity against *S. epidermidis* bacteria. Zone diameters and differences in zone sizes are listed in Table 6 and Figure 15, respectively. In the literature, it is indicated that the mineral content (Zn, Mg, Cu etc.), antimicrobial peptides (copper peptides), and achacin in mucus and slime secretions ensure antimicrobial properties.^22,45^ Dolashka et al. obtained six different peptides from *H. aspersa* mucus and investigated the antimicrobial activity of peptides against *Propionibacterium acnes*, *E. coli*, and *H. pylori* bacteria,^46^ as well as Pitt et al. proved the antimicrobial activity of snail mucus against *Pseudomonas aeruginosa*.^47^ Moreover, in our previous study, we observed clear zones around the discs for *S. epidermidis* and *P. fluorescence* bacteria.^20^ Overall, antimicrobial results indicated that snail extracts at this concentration range showed slight antimicrobial activity compared to conventional antibiotics. Also, in vitro release studies indicated that slime extract diffused from the genipin cross-linked chitosan nanofiber layer in a sustained manner but for a short period of 3–4 days. Therefore, snail extract loading and release in the nanofiber layer can be improved by using a nanoparticle-based carrier in the polymer matrix to enhance the antimicrobial activity of bilayer wound dressing.

**Table 6:** Inhibition Zone Diameters of Bilayer Wound Dressings’ Release Media

groups	inhibition zones of *S. epidermidis* (mm)
amoxicillin	9–13
control	
group 1	1 ± 0
group 2	0.917 ± 0.14

### 3.8 In Vitro Cell Culture Studies

#### 3.8.1 Cytotoxicity Analysis

The in vitro cytotoxicity of mucus and slime extracts was investigated using NIH/3T3 and HS2 cell lines to simulate the skin tissue response. The results indicated that mucus and slime extracts did not exhibit any toxic effects on fibroblast and keratinocyte cells (Figures 19b and 20b). Additionally, a high percentage of cell viability was achieved throughout the culture period in both NIH/3T3 and HS2 cell lines. Similarly, previous studies conducted by Trapella et al. and Gentili et al. demonstrated that *H. aspersa* mucus did not have cytotoxic effects on fibroblast and keratinocyte cells.^24,48^

#### 3.8.2 Wound Healing

**Figure 16:**
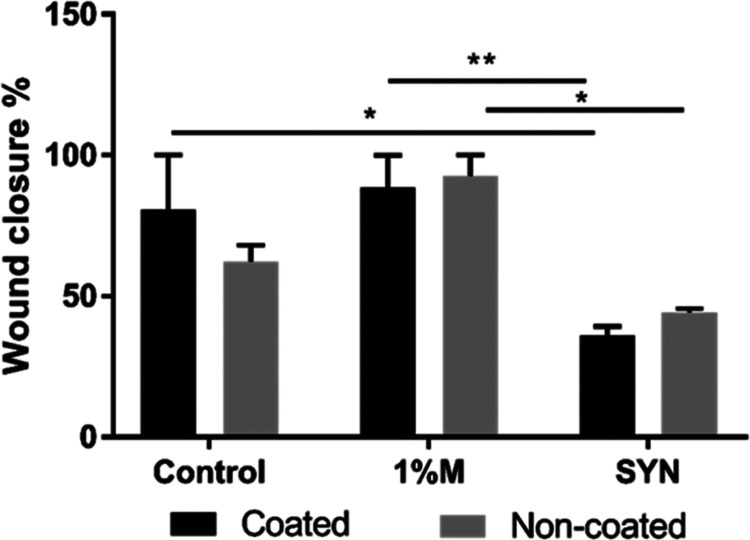
Wound closure % of fibroblast cells incubated with snail mucus and synergic extract media on coated and noncoated surfaces. (*p* < 0.05 is represented as *; *p* < 0.01 is represented as **).

**Figure 17:**
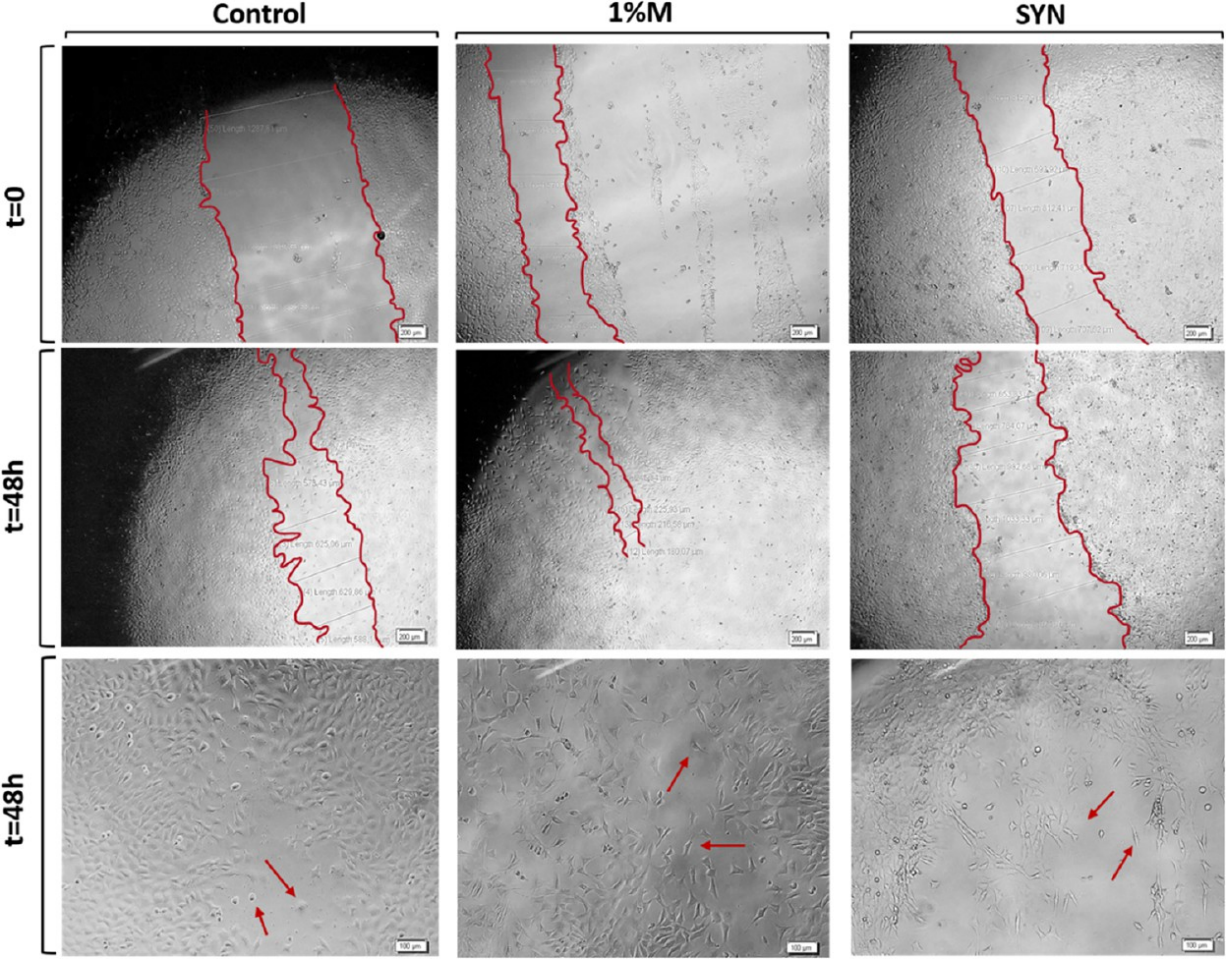
Microscopy images of wound boundaries of NIH/3T3 fibroblast cells in a 48 h incubation period on poly-l-lysine-coated surfaces for the control, 1%M, and SYN samples.

**Figure 18:**
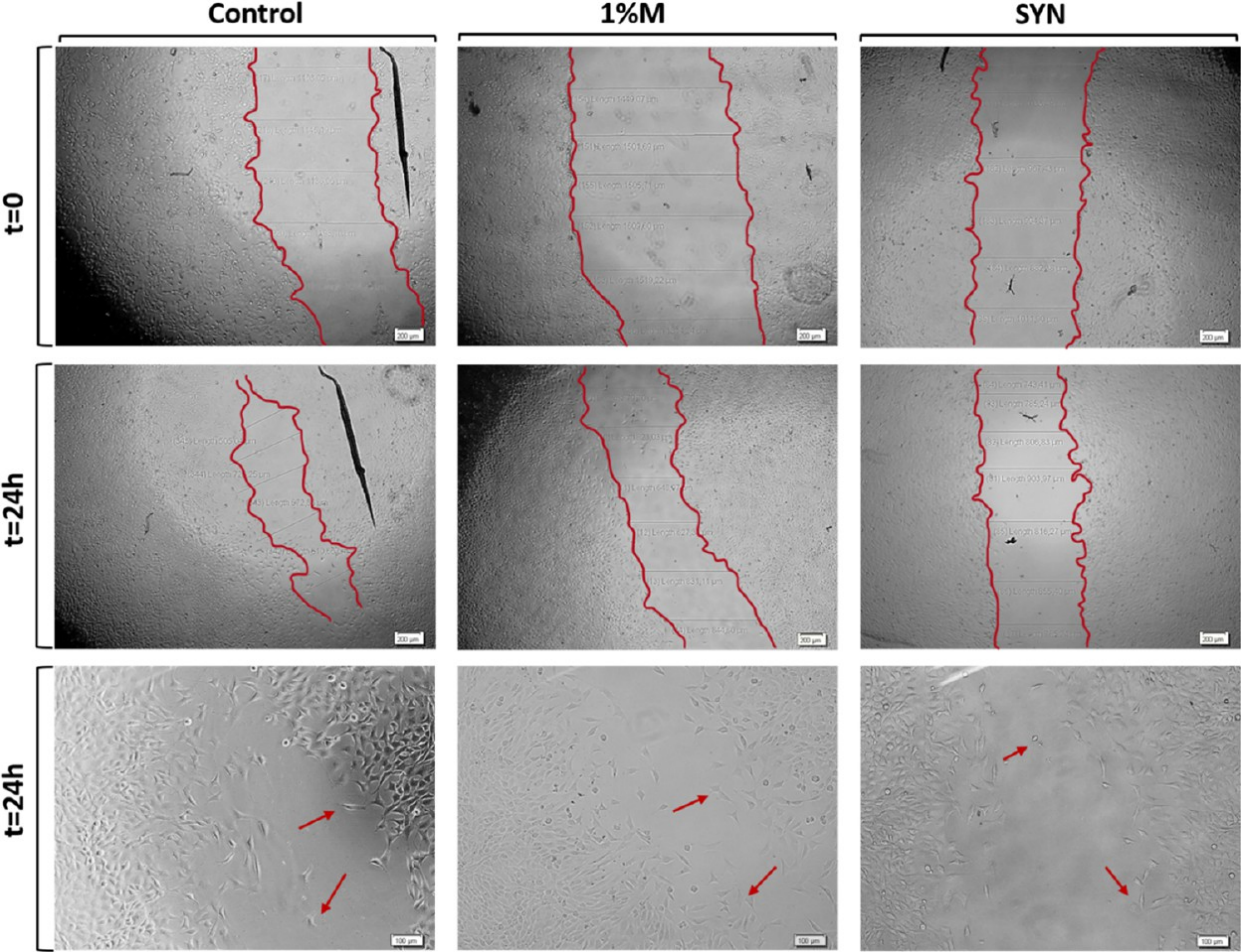
Microscopy images of wound boundaries of fibroblast cells in a 24 h incubation period on noncoated surfaces for the control, 1%M, and SYN samples.

The wound healing potential of snail mucus and slime extracts was investigated by using an in vitro scratch assay. The proliferation of NIH/3T3 fibroblast cells was assessed on noncoated 96-well plates, while the migration capacity was evaluated on poly-l-lysine-coated 96-well plates. The wound closure percentage data for the fibroblast monolayer treated with mucus and slime extraction media are depicted in Figure 16. Additionally, microscopy images depicted the migration (Figure 17) and proliferation (Figure 18) of NIH/3T3 fibroblast cells at the scratch area. Microscopy images revealed that fibroblast cells were positioned at the wound boundaries, proliferating and migrating into the wound gap. The results demonstrated that fibroblast cell migration was induced by snail mucus extraction media at 48 h compared to the control group. Furthermore, mucus and synergic extraction media (containing both mucus and slime extracts) enhanced fibroblast proliferation on noncoated surfaces at 24 h. By 48 h, NIH/3T3 cells were predominantly located at the wound boundary area for the synergic extract media on poly-l-lysine-coated surfaces. Overall, the results indicate that mucus extract induces fibroblast cell migration, while slime extract promotes fibroblast cell proliferation at the wound boundaries. The bioactive ingredients in mucus and slime (allantoin and glycolic acid) increase fibroblast cell proliferation, and mucin, copper proteins, and glycoproteins stimulate collagen synthesis. Thus, skin damage regeneration and wound healing process are induced and accelerated.^45,49^ Previous studies in the literature also proved that *H. aspersa* secretions induced cell proliferation and stimulated wound healing.^23,24,48^

#### 3.8.3 Cell Proliferation

**Figure 19:**
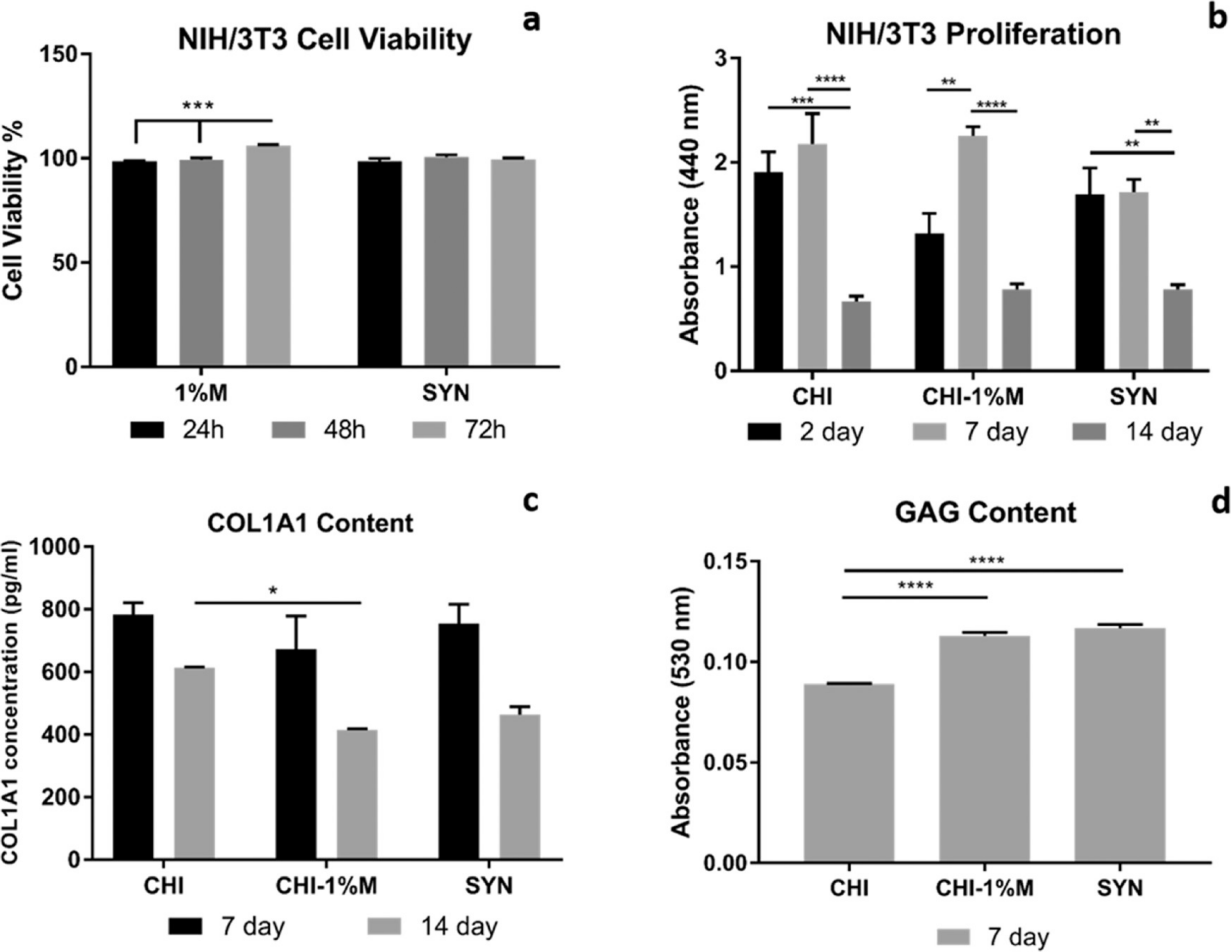
(a) Cell viability %, (b) cell proliferation, (c) collagen type I, and (d) GAG content for NIH/3T3 fibroblast cells on the spongeous layer of wound dressings (*p* < 0.1 is represented as *, *p* < 0.01 is represented as **; *p* < 0.001 is represented as ***; *p* < 0.0001 is represented as ****).

**Figure 20:**
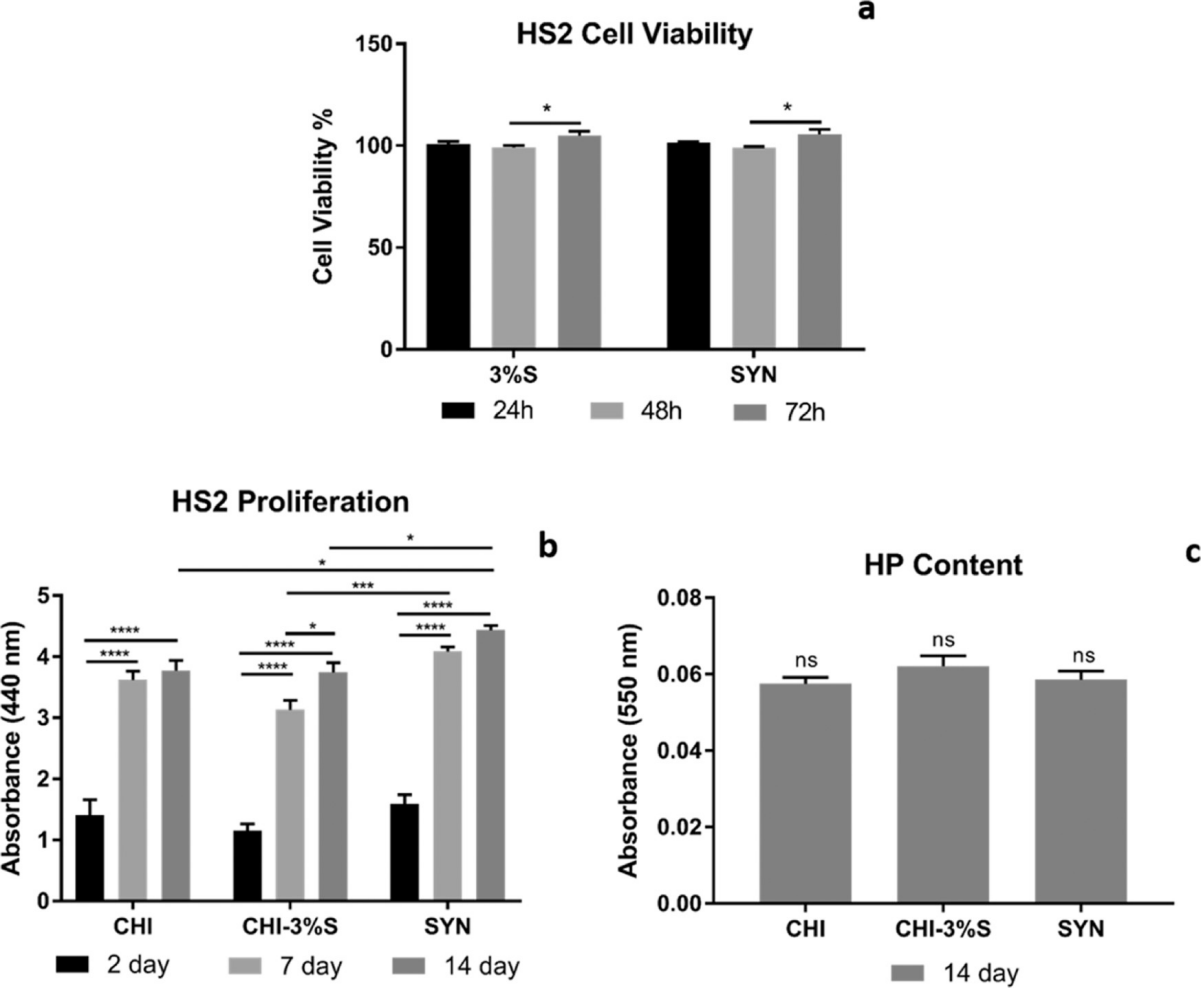
(a) Cell viability %, (b) cell proliferation, and (c) HP content for HS2 keratinocyte cells on nanofiber layers of wound dressings (*p* < 0.05 is represented as *; *p* < 0.001 is represented as ***; *p* < 0.0001 is represented as ****).

Cell proliferation of bilayer wound dressings was assessed with the WST-1 assay using NIH/3T3 cells for the spongeous layer and HS2 cells for the nanofiber layer separately over a period of 14 days (Figures 19b and 20b). The highest level of cell proliferation was observed for NIH/3T3 cells on the CHI–1%M spongeous layer at the 7th day compared to the SYN group. However, NIH/3T3 fibroblast cell proliferation showed a decreasing trend at the 14th day for all groups due to the mucoadhesive property of the extracts on the material surface, leading to cell colonization and preventing spreading. In addition, bioactive and similar components of mucus and slime extracts may induce the ECM matrix formation of fibroblast cells after 7 days of incubation rather than proliferation. HS2 keratinocyte cells highly proliferated on nanofiber groups up to 14 days of incubation. Among all groups, HS2 cell proliferation was found to be significantly higher on the SYN nanofiber layer at the 14th day of incubation. Snail mucus and slime secretions contain many bioactive compounds such as allantoin and glycolic acid, inducing cell growth and proliferation as well as the synthesis of ECM components.^45,50^ A literature study reported by Gentili et al. investigated the effect of mucus secretion on keratinocyte cells and found that mucus secretion significantly induced cell proliferation compared to the untreated group.^48^ Similarly, Angulo et al. demonstrated that mucus and aloe vera extract with incorporated gelatin/chitosan scaffolds induced fibroblast cell proliferation.^51^

#### 3.8.4 Cell Attachment and Spreading of Bilayer Wound Dressing

**Figure 21:**
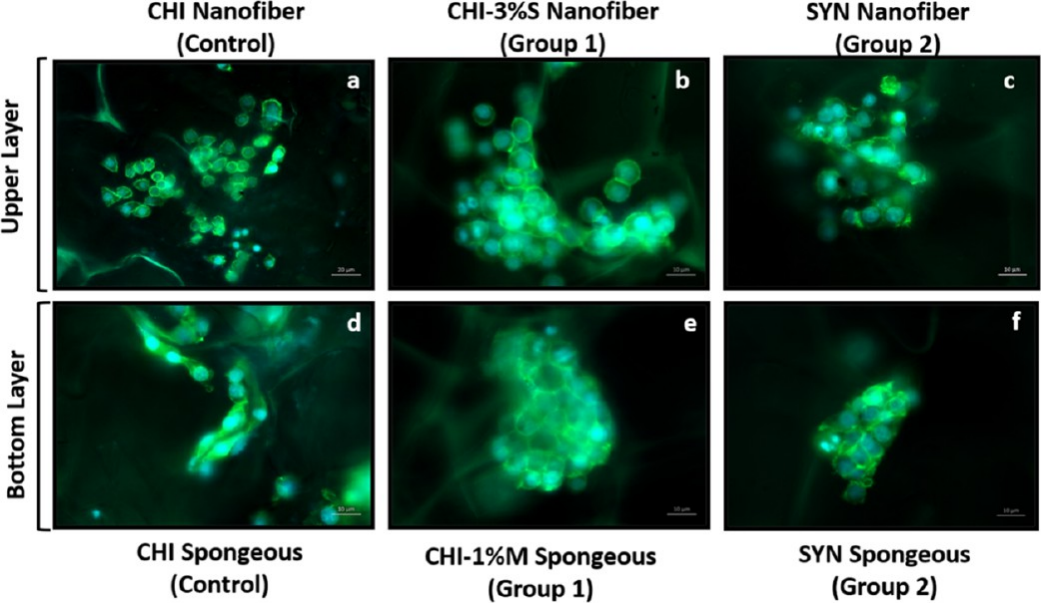
Fluorescence microscopy images for HS2 keratinocyte (a, b, c) and NIH/3T3 fibroblast (d, e, f) cell attachment on nanofiber and spongeous layers of wound dressings on the 3rd day, respectively (green and blue dyes represent actin/DAPI staining, 20 and 10 μm scale bars)

Attachment and spreading of NIH/3T3 and HS2 cells on the spongeous and nanofiber layers were assessed using fluorescence microscopy on the 3rd day (Figure 21). Fluorescence images revealed that both HS2 and NIH/3T3 cells successfully attached and displayed their characteristic morphology on the surface of both porous and nanofiber layers of the chitosan matrix (control), whereas fluorescence images depicted that HS2 and NIH/3T3 cells proliferated with colonized three-dimensional (3D) morphologies on group 1 and group 2 surfaces due to the mucoadhesive properties of snail secretion extracts. Similarly, Saos-2 and SW1353 cells attached and proliferated like colony forms on the material surface in our previous study for hard tissue applications.^20^

#### 3.8.5 Type I Collagen Secretion

Type I collagen is a crucial extracellular matrix (ECM) protein characterized by its triple helix structure composed of α_1_ and α_2_ chains.^52^ This unique structure provides integrity as well as biochemical and biomechanical strength to tissues such as bone and skin. Fibroblast cells within the dermis layer play a vital role in producing type I collagen, which is essential for tissue repair during the wound healing process.^53,54^ In our study, we examined the collagen type I content of fibroblast cells on porous layers at the 7th and 14th days of incubation. The results revealed that collagen deposition at the 7th day was similar across all groups, but statistically significant differences were observed between CHI and CHI–1% M groups at the 14th day (Figure 19c). Previous studies in the literature indicated that *H. aspersa* mucus and slime consist of glycolic acid and collagen as well as many glycoproteins and peptides (e.g., copper peptides) that stimulate collagen synthesis.^45,55^

#### 3.8.6 Hydroxyproline Content

Hydroxyproline, an amino acid consisting of 4-hydroxy-l-proline and 3-hydroxy-l-proline isomeric form^56^ found within collagen fibers, serves as a critical biomarker for the wound healing process. Elevated hydroxyproline content indicates increased collagen synthesis and cell proliferation, thereby promoting tissue repair and healing.^57,58^ In our study, we measured the hydroxyproline content of the upper layers after 14 days of incubation due to keratinocyte cells involving 3-hydroxyproline and 4-hydroxyproline ECM components.^59^ The results revealed that the hydroxyproline contents of all groups were similar, with no significant differences observed between them (Figure 20c).

#### 3.8.7 GAG Content and Safranin-O Staining

**Figure 22:**
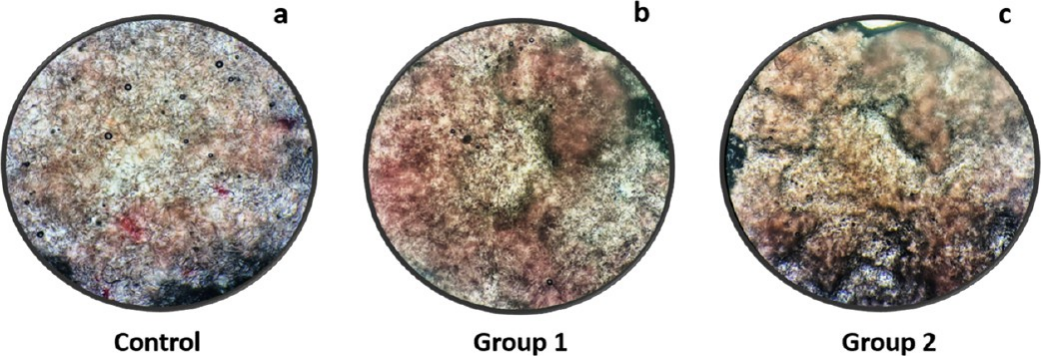
Microscopy images of glycosaminoglycan deposition on (a) the control group, (b) group 1, and (c) group 2 wound dressing surfaces with Safranin-O staining (10× magnification).

Glycosaminoglycans (GAGs) are polysaccharides found in the extracellular matrix (ECM), comprising repeating disaccharide units categorized into heparin/heparan sulfate (HS), chondroitin sulfate (CS)/dermatan sulfate (DS), keratan sulfate (KS), and hyaluronic acid (HA). They play pivotal roles in cell adhesion, proliferation, tissue mechanical strength, and wound repair.^60^ During the proliferation phase of wound healing, fibroblasts increase GAG synthesis in the wound area,^61^ with HA being the most abundant GAG type (75%), primarily provided by fibroblast cells in the dermal layer of skin tissue, supporting scarless wound repair.^62^ Given that *H. aspersa* snail secretions contain glycoproteins, such as achacin, as well as hyaluronic acid, proteoglycans, and glycosaminoglycans,^59^ our study focused on assessing the glycosaminoglycan (GAG) content of fibroblast cells within the spongeous layer. This evaluation was conducted using a spectrophotometric proteoglycan detection kit with 7 days of incubation. The results indicated increased GAG production by fibroblast cells for the CHI–1% M and SYN spongeous layers, likely due to the incorporation of mucus and slime extracts into the chitosan matrix (Figure 19d). Furthermore, bilayer wound dressings were stained with Safranin-O dye to determine GAG production for all materials and visualized using light microscopy. The results revealed GAG deposition in all groups, with a significant increase observed in GAG production induced by snail mucus and slime extract incorporation into the polymer matrix (groups 1 and 2) compared to the control group (Figure 22).

## 4 Conclusions

In this study, bilayer wound dressings loaded with *H. aspersa* secretions were successfully produced by using lyophilization and electrospinning methods. Material characterization confirmed that the extract-loaded dressings possessed suitable nanofiber diameter and pore size for the attachment of keratinocyte and fibroblast cells. Despite a decrease in swelling percentage due to the presence of snail secretion extracts, the bilayer wound dressings maintained favorable swelling behavior, as demonstrated in previous studies. Moreover, the addition of mucus and slime extracts in the polymer matrix increased the Young’s modulus of the spongeous layers.

Antimicrobial analysis revealed that the snail secretion extract itself and the release media from the bilayer samples exhibited moderate antimicrobial activity against *S. epidermidis*. In vitro release studies indicated that both slime-loaded group 1 and slime–mucus-loaded group 2 showed a burst release up to 6 h. However, a sustained release was obtained for group 1 up to 96 h, whereas group 2 showed a controlled release up to 72 h.

In vitro bioactivity assays demonstrated that mucus and slime extracts had no cytotoxic effect on NIH/3T3 and HS2 cells and promoted cell attachment and proliferation when loaded into a polymer matrix. Additionally, GAG and collagen syntheses were observed on the surfaces of the bilayer materials, with significantly enhanced GAG secretion observed in *H. aspersa*-loaded scaffolds (groups 1 and 2) compared to the control group. Furthermore, in vitro wound healing assays confirmed that the addition of mucus and slime extracts induced cell proliferation and migration in the wound area. In conclusion, bilayer wound dressings loaded with bioactive mucus and slime extracts exhibit promising potential for wound healing applications.
